# Downscaling the ocean response to the Madden–Julian Oscillation in the Northwest Atlantic and adjacent shelf seas

**DOI:** 10.1007/s00382-024-07233-y

**Published:** 2024-05-06

**Authors:** Christoph Renkl, Eric C. J. Oliver, Keith R. Thompson

**Affiliations:** 1 Physical Oceanography Department, Woods Hole Oceanographic Institution, 266 Woods Hole Rd, Woods Hole, MA 02543 USA; 2 Department of Oceanography, Dalhousie University, 1355 Oxford St, Halifax, NS B3H 4R2 Canada

**Keywords:** Subseasonal-to-seasonal prediction, Madden–Julian oscillation, Teleconnections, Dynamical downscaling

## Abstract

Subseasonal-to-seasonal (S2S) prediction is a global effort to forecast the state of the atmosphere and ocean with lead times between two weeks and a season. This study explores the feasibility of S2S prediction of the ocean using a variety of tools including statistical analysis, a statistical-dynamical mixed layer model, and a regional, high-resolution ocean circulation model based on physical principles. Ocean predictability on S2S timescales is analyzed by compositing winter sea surface temperature (SST) anomalies in the North Atlantic with respect to the state of the Madden–Julian Oscillation (MJO). It is found that statistically significant, large-scale SST changes, particularly along the eastern seaboard of North America, can be related to the MJO. This signal is shown to be driven by anomalous air–sea heat fluxes caused by atmospheric perturbations in response to the MJO. The high-resolution model of the Gulf of Maine and Scotian Shelf is used to downscale the mean ocean response to the MJO. The model is able to capture the observed relationship between the MJO and SST in the northwest Atlantic. It is also shown that the anomalous atmospheric circulation in response to the MJO leads to anomalous upwelling on the Scotian Shelf. Overall, this study demonstrates that it is feasible, and of value, to use regional ocean models for S2S prediction.

## Introduction

Subseasonal-to-seasonal (S2S) prediction is a global effort to forecast the state of the atmosphere and ocean with lead times between two weeks and a season (Vitart and Robertson 2019). These predictions can provide valuable, early information for decision makers to ensure public safety, energy security, and protection of marine infrastructure (e.g., White et al. 2017; DeMott et al. 2021). Significant advances in S2S prediction have been made by the atmospheric community. While it is recognized that the ocean directly influences the atmospheric predictability on S2S timescales, less is known about the predictability of the ocean on such timescales (e.g., DeMott et al. 2015; Saravanan and Chang 2019; Merryfield et al. 2020; Amaya et al. 2022, and references therein).

S2S prediction relies considerably on teleconnections as a source of predictability which can often be traced back to the tropics (Lin et al. 2019). On S2S timescales, variability in the tropics is dominated by the Madden–Julian Oscillation (MJO, Madden and Julian 1971, 1972) which can influence the atmospheric circulation around the globe (e.g., Zhang 2013; Woolnough 2019). The MJO is a large-scale convective anomaly in the atmosphere that propagates eastward along the equator with a period of 40–60 days. Upward motion associated with the deep convection near the equator leads to divergent outflow aloft creating a source for Rossby waves that propagate poleward and eastward (Sardeshmukh and Hoskins 1988). Consequently, a wave train is established influencing the extratropical circulation (e.g., Matthews et al. 2004; Seo et al. 2012; Seo and Lee 2017; Stan et al. 2018). It has been shown that this teleconnection is most effective when the MJO has a dipole structure with enhanced convection over the Indian Ocean and suppressed convection over the western Equatorial Pacific, and vice versa (Lin et al. 2010). Most of the known MJO teleconnections are documented for boreal winter (Stan et al. 2018) when the MJO activity is stronger and the westerly waveguide is not interrupted (e.g., Zhang and Dong 2004; Adames et al. 2016).

In the extratropics, the coherent circulation anomalies related to the MJO interact with modes of climate variability and have direct influence on weather (see Stan et al. 2018, for a comprehensive review). For example, Cassou (2008) and Lin and Brunet (2009) showed that the North Atlantic Oscillation (NAO) is more likely to occur in its positive and negative phase about 5–15 days after the MJO-related convection reaches the eastern Indian Ocean and western Equatorial Pacific, respectively. The MJO has also been shown to influence surface air temperature in the northern hemisphere (e.g., Vecchi and Bond 2004; Lin and Brunet 2009; Zhou et al. 2012; Baxter et al. 2014; Seo et al. 2016; Hu et al. 2019) and precipitation across North America (e.g., Lin et al. 2010; Jones and Carvalho 2012; Baxter et al. 2014; Klotzbach et al. 2016). This can ultimately lead to extreme events like heatwaves and flooding related to the MJO.

The influence on the extratropical ocean has received relatively little attention (e.g., DeMott et al. 2021; Amaya et al. 2022). The ocean and atmosphere exchange heat and momentum through fluxes across the air–sea interface. It is therefore likely, that the atmospheric anomalies in response to the MJO also affect the surface ocean. Marshall et al. (2015) showed that outside the tropics, the MJO has an impact on ocean wind wave characteristics in the North Pacific and North Atlantic. They conclude that zonal wind anomalies related to the NAO response to the MJO lead to anomalies in significant wave height in the eastern North Atlantic. However, they also point out that some anomalous wave conditions occur before the NAO pattern is established in the atmosphere.

In this study, we explore the feasibility of S2S predictions of the ocean with particular focus on the Northwest Atlantic and adjacent shelf seas using observations and a hierarchy of statistical and physically based models. Section 2 gives a description of the Real-Time Multivariate MJO (RMM) index and the observations of sea surface temperature (SST) and air–sea heat fluxes. In Sect. 3, a composite analysis is applied to identify the mean response of both SST and net heat fluxes in the North Atlantic. A simple surface mixed layer model is used in Sect. 4, to explore if air–sea heat flux variations are a significant driver of SST variability on S2S timescales. It is shown that statistically significant, large-scale changes of observed winter SST in the northwest Atlantic can be related to anomalous heat fluxes in response to the MJO. Based on the identified temporal “windows of opportunity” of enhanced S2S predictability, in Sect. 5, we use a high-resolution regional ocean model of the Gulf of Maine and Scotian shelf region to dynamically downscale the ocean response to the MJO. This allows us to (1) identify the ocean response at smaller scales, (2) analyze how the atmospheric forcing in response to the MJO is projected into the subsurface ocean, and (3) demonstrate the feasibility of using such models for ocean predictions on S2S timescales. Section 6 summarizes the results and implications for S2S prediction of the ocean are discussed.

## Data

### Real-Time Multivariate MJO (RMM) index

For the purpose of this study, the state of the MJO is characterized by the daily Real-Time Multivariate MJO (RMM) index (Wheeler and Hendon 2004) obtained from the Australian Bureau of Meteorology. This index is based on satellite observations of outgoing longwave radiation (OLR) and reanalysis fields of zonal wind at 850 hPa and 200 hPa in the tropics (15 ${}^{\circ }$S–15 ${}^{\circ }$N). The bivariate MJO index is defined by the principal components associated with the first two combined empirical orthogonal functions (EOFs) referred to as RMM1 and RMM2. Due to the quasi-periodicity of the MJO, the RMM index is strongly autocorrelated. The autocorrelation function of the complex RMM index $(\text {RMM1} + i~\text {RMM2})$ is 0.3 at a lag of 20 days.

The amplitude of the RMM index is given by

1 $$\begin{aligned} A_{\text {RMM}}(t) = \sqrt{\text {RMM1}^2(t) + \text {RMM2}^2(t)} \end{aligned}$$

and the MJO is considered to be active when $A_\text {RMM} >{1}$. The angle

2 $$\begin{aligned} \phi _{\text {RMM}}(t) = \tan ^{-1} \left( \frac{-\text {RMM2}(t)}{\text {RMM1}(t)} \right) \end{aligned}$$

is typically quantized into eight integer phases $\Phi _{\text {RMM}}$ which describe the geographic position of the convective anomaly associated with the MJO. On average, the MJO stays in one phase for 6 days (Wheeler and Hendon 2004).

Daily values of the RMM index are available continuously from 1979 to present. In this study, we focus on boreal winter months (December through February, DJF) for the period 1981–2019.

**Fig. 1:**
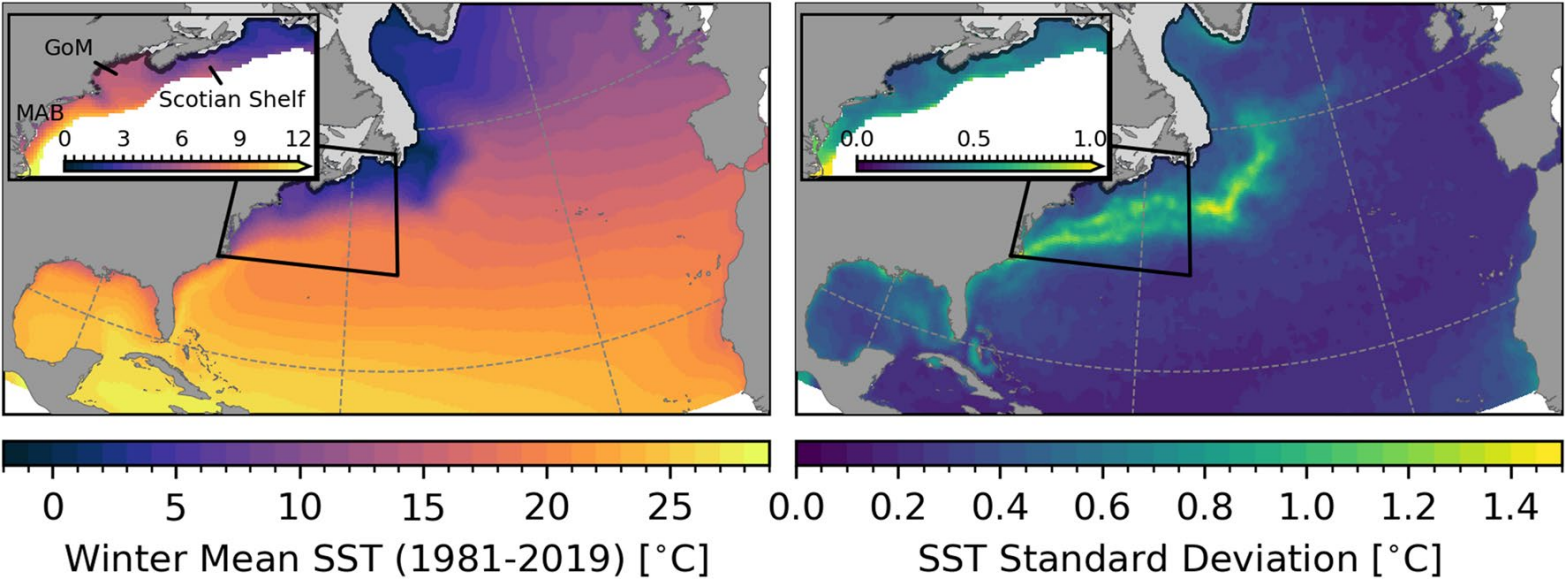
Winter SST in the North Atlantic. OISSTv2 winter mean SST (left), and standard deviation of bandpass-filtered SST anomalies $T_\text {a}(\textbf{x}, t)$ for the period 1981–2019 (right). The insets show an enlarged view of the Middle Atlantic Bight (MAB), Gulf of Maine (GoM), and Scotian Shelf regions indicated by the black quadrangle. In these panels, values are only shown at grid points with water depth less than or equal to 1000 m. The light area near the coast shows the climatological sea ice cover

### NOAA Optimal Interpolation Sea Surface Temperature

Version 2.0 of the Optimal Interpolation Sea Surface Temperature dataset (OISSTv2, Reynolds et al. 1981) produced by the National Oceanic and Atmospheric Administration (NOAA) provides statistically interpolated satellite observations of SST supplemented with in-situ measurements. Daily SST values are available on a global grid with 0.25 ${}^{\circ }$ spacing from September 1981 to present. In this study, the focus is on the North Atlantic region between 15 ${}^{\circ }$ to 65 ${}^{\circ }$N and 262 ${}^{\circ }$ to 360 ${}^{\circ }$E for the period from December 1981 to February 2019, covering 37 full winter seasons (DJF). In the following, these daily SST fields will be denoted by $T(\textbf{x}, t)$, where $\textbf{x}$ is the location vector of a given grid cell and *t* is time.

At each grid point in the study area, the climatology $T_\text {c}(\textbf{x}, t)$ was computed using a methodology similar to that of Hobday et al. (2016). First, the mean for each day of the year is calculated over the climatology period (here 1982–2018) and then a running mean with an 11-day window centered around the day of interest is applied. (Note that year end effects are taken into account, e.g., the climatological value for January 1 is the mean value over the period from December 27 to January 6 over all years). For leap years, the value for February 29 is computed as the arithmetic mean of the values for 28 February and 1 March.

Anomalies were computed by subtracting the smoothed daily climatology from the daily SST fields

3 $$\begin{aligned} T_\text {a}(\textbf{x}, t) = T(\textbf{x}, t) - T_\text {c}(\textbf{x}, t). \end{aligned}$$

In order to extract the SST variability on S2S timescales, a third order digital Butterworth bandpass filter (Butterworth 1930) with a 15- to 100-day passband was applied to the SST anomaly time series at each grid point. For the remainder of the study, $T_\text {a}(\textbf{x}, t)$ refers to the bandpass-filtered SST anomalies.

Figure 1 shows the winter mean SST and standard deviation of $T_\text {a}(\textbf{x}, t)$ for the whole study area and as an enlarged view of the Middle Atlantic Bight, Gulf of Maine, and Scotian Shelf (insets). The dominant feature in the domain is the Gulf Stream indicated by strong gradients and high variability in SST. Note that the regions with a high standard deviation are confined to the deep ocean.

### Objectively Analyzed Air–Sea Fluxes (OAFlux)

Gridded fields of turbulent heat fluxes between the ocean and atmosphere are taken from the third version of Objectively Analyzed Air–Sea Fluxes (OAFlux, Yu and Weller 2007). This dataset is a synthesis of satellite observations and reanalysis products of sea surface temperature *T*, air temperature $T_{\text {air}}$, specific humidity $q_{\text {air}}$ at 2 m, and wind speed $U_\text {wind}$ at 10 m. Each field of these variables was computed independently using variational objective analysis to yield the best estimates in a least squares sense. From these fields, air–sea heat fluxes were computed using the Coupled Ocean–Atmosphere Response Experiment algorithm (COARE, Fairall et al. 2003) based on following bulk formulae for sensible heat flux

4 $$\begin{aligned} Q_\text {s} = \rho _\text {a} c_{p,\text {a}} c_\text {h} U_\text {wind} \left( T - T_{\text {air}} \right) \end{aligned}$$

and latent heat flux

5 $$\begin{aligned} Q_\text {l} = \rho _\text {a} c_\text {e} L_\text {e} U_\text {wind} \left( q_\text {s} - q_{\text {air}} \right) , \end{aligned}$$

where $\rho _\text {a}$ is the density of air, $q_\text {s}$ is the saturation humidity at *T* taking into account the reduced vapor pressure caused by salt water (Yu 2009), and $c_{p,\text {a}}$ is the specific heat capacity of air at constant pressure; $c_\text {h}$ and $c_\text {e}$ are the turbulent exchange coefficients for latent and sensible heat, respectively, and $L_\text {e}$ is the latent heat of evaporation.

Daily mean fields of turbulent air–sea heat fluxes are available for the period 1985–2019 on a global grid with 1 ${}^{\circ }$ spacing. In addition, surface radiation data of incoming shortwave $Q_\text {sw}$ and outgoing longwave radiation $Q_\text {lw}$ from the International Satellite Cloud Climatology Project (ISCCP, Zhang et al. 2004) as well as net heat flux

6 $$\begin{aligned} Q_{\text {net}} = Q_\text {sw} - Q_\text {lw} - Q_\text {s} - Q_\text {l}. \end{aligned}$$

were obtained on the same grid from the OAFlux database. This equation reflects the sign convention of the OAFlux dataset. The daily mean radiation and $Q_{\text {net}}$ data are only available for the years 1985–2009 and therefore, the heat flux analysis in this study is limited to this period. Anomalies of the heat flux components were calculated in the North Atlantic region the same way as for SST. The same Butterworth bandpass filter was applied to extract the heat flux variability on S2S timescales.

## Composite analysis of observed SST and net heat flux

The relationship between SST of the North Atlantic and the MJO is analyzed using a composite analysis. Consider the bandpass-filtered SST anomaly at a fixed location $T_\text {a}(\textbf{x}, t)$ to be a random variable of a stationary process. The conditional mean (e.g., Priestley 1981) of $T_\text {a}$ at lag $\delta$ is defined by

7 $$\begin{aligned} \mu (\textbf{x}, j, \delta ) = \text {E} \left[ T_\text {a}(\textbf{x}, t + \delta ) | \Phi _{\text {RMM}}(t) = j, A_{\text {RMM}}(t) > 1 \right] . \end{aligned}$$

Here, $\text {E}[\cdot ]$ is the expectation operator which is applied to the subset of $T_\text {a}$ defined by the time when the MJO phase $\Phi _{\text {RMM}} = j$, where $j =$ 1, 2,..., 8, and $A_\text {RMM}>$ 1. It is clear from (7) that the conditional mean is a function of location ($\textbf{x}$), MJO phase (*j*), and lag ($\delta$).

For each combination of $\Phi _{\text {RMM}} = j$ and $\delta$, the conditional means were calculated using  (7) where the expectation operator was replaced by the sample mean. These estimates will henceforth be referred to as composites and denoted by $\overline{T}_\text {a}$. The maximum lag considered in this study is $\delta =$ 42 days which approximately corresponds to the time it takes the MJO to propagate through seven phases. This means the lagged composites also contain data for the months of March and April. It is important to recognize that the samples in each composite are not independent because they can be from consecutive days and therefore are autocorrelated.

In order to test if a composite is significantly different from zero, a moving-blocks bootstrap is applied following Henderson et al. (2016). This significance test preserves the autocorrelation structure of the samples in each composite. For each MJO phase, *N*/6 potentially overlapping 6-day blocks of the full record of $T_\text {a}$ are randomly selected with replacement where *N* is the number of samples in each composite. These blocks constitute a bootstrap sample with the same number of days as the MJO composite expected for a given phase. The length of the blocks was chosen based on the average time the MJO spends consecutively in each phase. This process was repeated 1000 times and the mean of each bootstrap sample was computed. From the 1000 bootstrap means, the quantiles of the underlying probability distribution were estimated and then compared to $\overline{T}_\text {a}$. Here, the composited anomalies are considered statistically different from zero at the 10% significance level if they are below or above the 5th and 95th percentile of the bootstrap distribution, respectively.

**Fig. 2:**
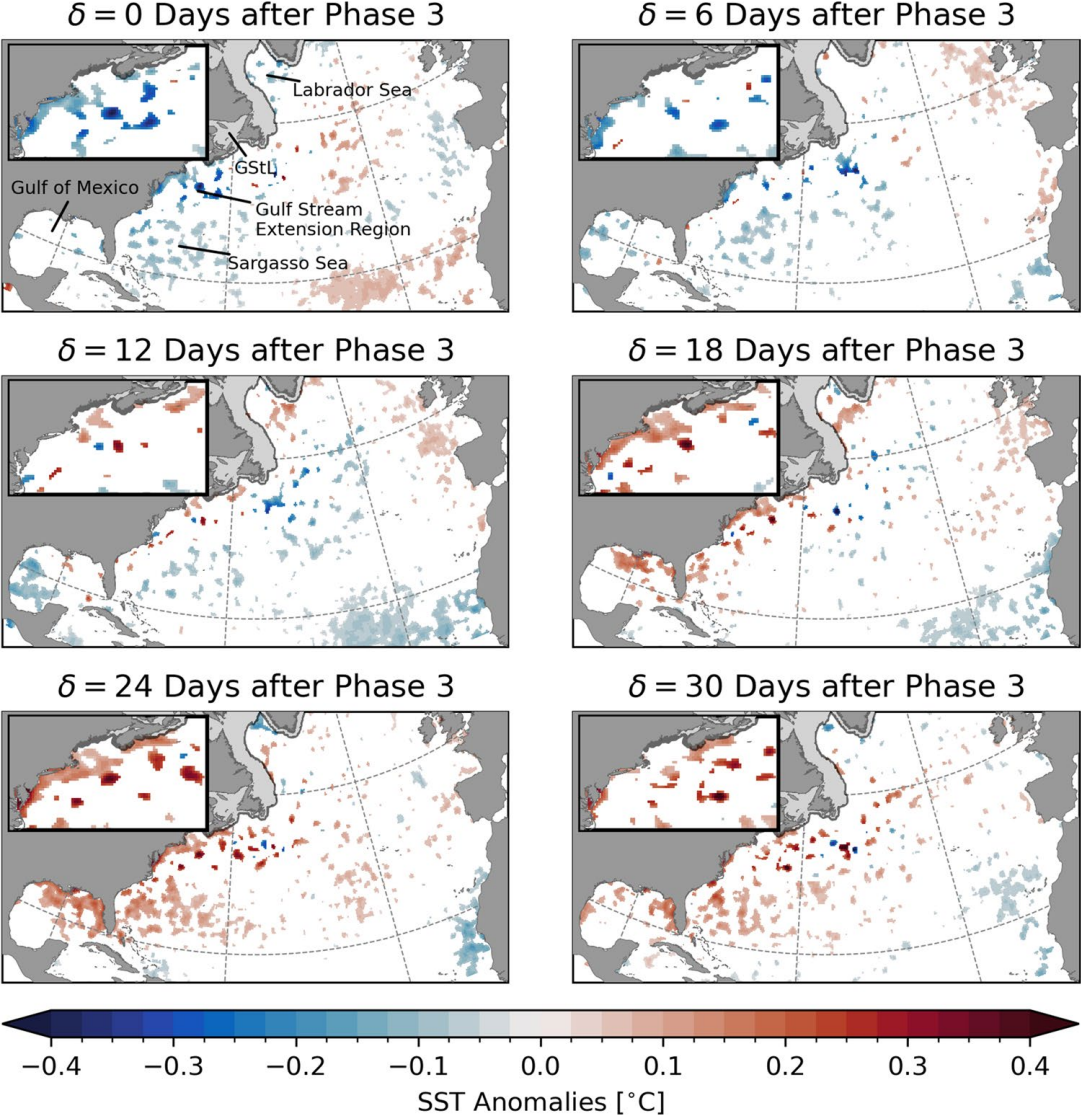
Composites of bandpass-filtered SST anomalies $\overline{T}_\text {a}$ for lags $\delta =$ 0, 6, ..., 30 days after MJO phase 3 during winter when $A_\text {RMM}>$ 1. Maps show the spatial structure of the composites for the whole study area and in the Middle Atlantic Bight, Gulf of Maine, and Scotian Shelf region (insets). Only anomalies statistically different from zero at the 10% significance level are shown. Shaded areas near the coast show the climatological sea ice cover

**Fig. 3:**
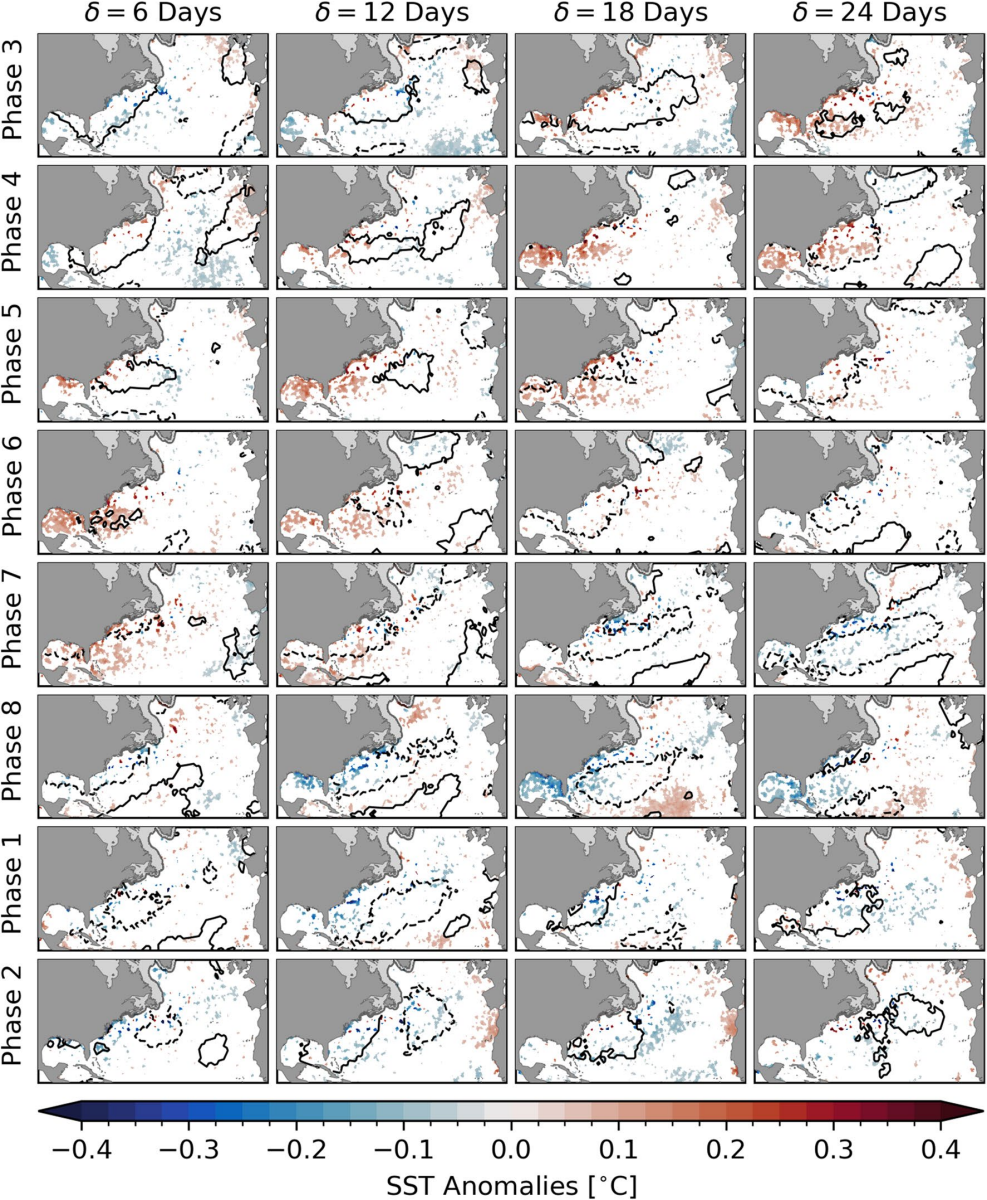
Composites of SST (colors) and $Q_{\text {net}}$ (contours) with respect to all MJO phases during winter when $A_\text {RMM}>$ 1. Areas of significantly increased and reduced net heat flux from the atmosphere into the surface ocean are marked by solid and dashed contours, respectively. Each row represents a specific phase and the columns refer to lags $\delta =$ 6, 12, 18, and 24 days after that phase. All shown anomalies are statistically different from zero at the 10% significance level. Shaded areas near the coast show the climatological sea ice cover

Figure 2 shows the composites $\overline{T}_\text {a}$ with respect to MJO phase 3 that has previously been shown to lead to increased near-surface air temperature over the eastern US and Canada (e.g., Lin and Brunet 2009; Baxter et al. 2014). Map insets show an enlarged view of the Middle Atlantic Bight, Gulf of Maine, and Scotian Shelf region. Only composites statistically different from zero at the 10% significance level are shown. The shaded areas in the Gulf of St. Lawrence and western Labrador Sea are mostly covered by sea ice during the winter months (sea ice concentration greater than 15% during at least 90% of days in DJF) and are therefore excluded from the analysis.

The contemporaneous composite ($\delta =$ 0 days) shows a cold anomaly along the eastern seaboard of North America with significant anomalies in shallow areas in the Middle Atlantic Bight. In the offshore, weak and scattered negative anomalies occur in the Sargasso Sea. Additionally, a widespread increase in SST of about 0.1 ${}^{\circ }\text {C}$ can be observed in the tropical North Atlantic.

The significant anomalies in the Middle Atlantic Bight, Gulf of Maine, and Scotian Shelf region only occur on the continental shelf. The largest response occurs in Chesapeake Bay and Delaware Bay which are shallow estuaries with water depths $\mathcal {O}$(20 m). Composite maps disregarding the statistical significance of the anomalies show a large-scale response extending to the deep ocean (not shown). However, the mesoscale eddy variability in the Gulf Stream Extension Region (Fig. 1) increases the background noise level masking the response to the MJO.

With increasing lag, the anomalies described above become weaker. After about 2 weeks, a warm anomaly develops in the western North Atlantic and Gulf of Mexico reaching its maximum 18–24 days after phase 3 depending on the location. The widespread anomaly is $\mathcal {O}$(0.2 ${}^{\circ }\text {C}$) with the strongest response in the Middle Atlantic Bight reaching up to 0.4 ${}^{\circ }\text {C}$. During the same time, a widespread cold anomaly occurs in the eastern tropical regions.

Overall, this demonstrates that statistically significant, large-scale changes of observed winter SST of the North Atlantic can be related to the MJO with the strongest response occuring on the shelf along the eastern seaboard of North America. It is expected that the extratropical response occurs at some lag with respect to the time when the MJO reaches a phase that is favourable for teleconnections (e.g., Cassou 2008; Henderson et al. 2016). Note that the lag at which the maximum ocean response to the MJO occurs can, conceptually, be considered the sum of two components:

8 $$\begin{aligned} \delta = \delta _\text {T} + \delta _\text {O}, \end{aligned}$$

where $\delta _\text {T}$ is the time for the atmospheric signal to propagate from the tropics to the North Atlantic region and $\delta _\text {O}$ is the timescale for the surface ocean to respond to an atmospheric perturbation. This can include the effects of turbulent heat exchange at the sea surface and advection by ocean currents. The teleconnection timescale of the atmosphere $\delta _\text {T}$ is on the order of 1–2 weeks (e.g., Cassou 2008; Lin and Brunet 2009; Lin et al. 2009; Baxter et al. 2014; Lin et al. 2019). A similar timescale is plausible for the upper ocean response (e.g., Deser and Timlin 1997) which leads to a total lag of $\delta =$ 2–4 weeks in agreement with the results of the composite analysis above. Note that, based on the average time the MJO spends in a particular phase (6 days), the total lag $\delta =$ 18–24 days corresponds to roughly half a canonical cycle. Given this delay, it is likely that the anomalies occurring at $\delta =$ 0 days are a lagged response to the MJO phase half a cycle before, i.e., phase 7.

The results presented in Fig. 2 illustrate the relationship between the SST and phase 3 of the MJO. To show the mean ocean response to a full MJO cycle, we now calculate SST composites with respect to all eight phases (Fig. 3). The first row in Fig. 3 shows the same maps as in Fig. 2. Each row represents a phase and the columns refer to lags of $\delta =$ 6, 12, 18, and 24 days. Note that the composites for $\delta =$ 0 days are not presented here because they are assumed to indicate a lagged response to a previous phase. It is important to point out the similarity of the anomaly patterns in the panels along the diagonals. This demonstrates a known issue: the quasi-periodicity of the MJO creates an ambiguity between MJO phases and the time lag at which the teleconnection occurs (e.g., Jenney et al. 2019).

The warm anomaly in the western North Atlantic and in the Gulf of Mexico developing 2–3 weeks after phase 3 can also be seen at shorter lags with respect to phases 4 to 7. Note that this anomaly appears to be more pronounced at $\delta =$ 18 days after phase 4. Based on the mean period of the MJO, this timing is in agreement with the composite 24 days after phase 3 described above because, on average, the MJO spends six consecutive days in one phase before it moves to the next. Therefore, a relationship with a particular phase and lag appears to be similar to the relationship of the next phase with $\delta$ about 6 days shorter. However, this duration varies and it is possible that the MJO skips a phase or decays before completing a full cycle. Thus, the composites along the diagonals are not completely identical.

The same issue occurs for the other half of the MJO cycle. At $\delta =$ 18 days after phase 8, significantly lower SST can be observed along the North American east coast and in the Gulf of Mexico. In terms of offshore extent, this cold anomaly is unique compared to the other phases and lags, however, in coastal regions, a similarity of the response along the diagonals is apparent. This shows again that the anomalies at $\delta = 0$ with respect to phase 3 shown in Fig. 2 are a delayed response.

Although the SST composites are generally small (< 0.5 ${}^{\circ }\text {C}$), it is important to note that they present a climatological average and the response to individual MJO episodes is expected to be much stronger. Due to the varying propagation speed of the MJO and response time, the averaging of multiple impulses leads to a further attenuation of the signal. Additionally, the spatial smoothing applied during the statistical interpolation of OISSTv2 (see Reynolds et al. 1981) as well as the filtering of the data will also attenuate the SST signal. The same composite analysis with the SST observations conservatively regridded on a grid with 1 ${}^{\circ }$ spacing did not change the results appreciably (not shown). Given the ambiguity in the lag due to the quasi-periodicity of the MJO, and the multiple timescales involved, it is not straightforward to quantify the relationship between a single MJO episode and the resulting SST anomalies.

The spatial scales of the SST composites suggest large-scale atmospheric anomalies to drive the observed variations in the surface ocean. We performed the same composite analysis on net heat fluxes in the North Atlantic (see Appendix). In Fig. 3, solid contours show areas of significantly increased heat flux and dashed lines mark regions of reduced heat flux from the atmosphere into the surface ocean in response to the MJO. Generally, the sign and spatial structure of the heat flux composites is similar to the SST response at longer lags. This difference in lags can be explained by the additional timescale $\delta _\text {O}$ for the SST to respond to the anomalous heating or cooling. To further explore this physical mechanism, we apply a surface mixed layer model that predicts SST variations in response to forcing by the observed net heat flux.

## Statistical-dynamical modelling of large-scale North Atlantic SST anomalies

SST anomalies can be considered an integrated response to the net air–sea heat flux into the ocean. In the following, we use a surface mixed layer model to test if observed air–sea heat flux variations related to the MJO are a significant driver of winter SST variability on S2S timescales for the North Atlantic.

**Fig. 4:**
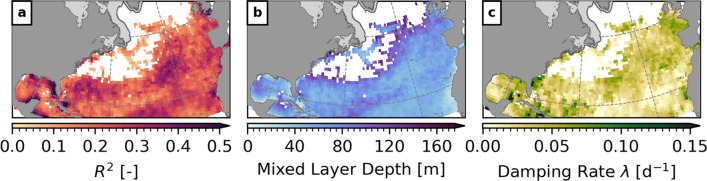
Estimated parameters and fit of the surface mixed layer model during winter. **a** Measure of model fit $R^2$ defined by (13). **b** Mixed layer depth $H_\text {m}$ in meters. **c** Damping rate $\lambda$ in day^−1^. Note that the color scales have been clipped. Shaded areas near the coast show the climatological sea ice cover. Results are only shown at grid points where $R^2$ is statistically different from zero at the 5% significance level

### Surface mixed layer model

A simple one-dimensional surface mixed layer heat budget can be written (e.g., Lagerloef et al. 1998)

9 $$\begin{aligned} \frac{\partial T_\text {a}}{\partial t} = \frac{Q_{\text {net}} - rQ_\text {sw}}{\rho _{0} c_{p,\text {w}} H_\text {m}} + M, \end{aligned}$$

where $T_\text {a}$ is the vertically averaged temperature anomaly in the mixed layer with depth $H_\text {m}$, $Q_{\text {net}}$ is the net air–sea heat flux anomaly (positive downwards), and *r* is the fraction of incoming shortwave radiation $Q_\text {sw}$ that reaches the base of mixed layer. $\rho _{0} =$ 1026 kg m$^{-3}$ and $c_{p,\text {w}} =$ 3985 Jkg$^{-1}$K$^{-1}$ are the density and specific heat capacity of seawater, respectively. Other ocean processes, e.g., horizontal advection, vertical mixing, or entrainment, that cause dissipation of heat from the mixed layer are summarized by *M*.

During winter, the mixed layer in the North Atlantic is assumed to be deep enough that the penetrating $Q_\text {sw}$ is negligibly small and therefore $r = 0$. The processes in *M* are parameterized as $-\lambda T_\text {a}$. They include the effects of horizontal and vertical diffusion of $T_\text {a}$ in restoring the temperature back to its climatology. As a result, (9) can be approximated by a first-order autoregressive model (e.g., Lagerloef et al. 1998)

10 $$\begin{aligned} \frac{\partial T_\text {a}}{\partial t} = \frac{Q_{\text {net}}}{\rho _{0} c_{p,\text {w}} H_\text {m}} - \lambda T_\text {a}, \end{aligned}$$

Note that (10) does not include advection which could also lead to SST anomalies and may play a role on S2S timescales.

Because the net heat flux associated with synoptic variability of the atmosphere has a shorter characteristic timescale in comparison to SST anomalies, $Q_{\text {net}}$ can be considered as stochastic forcing of the surface ocean. The constant damping rate $\lambda > 0$, which has units time^−1^, causes the model to have a statistically stationary response to stationary stochastic forcing (Frankignoul and Hasselmann 1977). The response to an impulse is an exponential decay with an *e*-folding time $\lambda ^{-1}$. If $Q_{\text {net}}$ is purely stationary, the absence of dissipation ($\lambda = 0$) would lead to a linear increase of the SST anomaly variance over time. On the other hand, if $\lambda \rightarrow \infty$, any input of heat is rapidly dissipated and the predicted SST anomaly would tend to zero.

Following Lagerloef et al. (1998), using a simple, explicit forward differencing scheme, the discrete form of (10) becomes

11 $$\begin{aligned} T_\text {a}^{t+1} = \frac{Q_{\text {net}}^{t}}{\rho _{0} c_{p,\text {w}} H_\text {m}} \Delta t + \left( 1 - \lambda \Delta t \right) T_\text {a}^{t}, \end{aligned}$$

where $\Delta t$ is the time difference between the model time steps denoted by superscript $t = 0, 1,..., N_{}$. Here, we use $\Delta t =$ 1 day.

Based on the assumption that the temperature is constant throughout the surface mixed layer, the model in (11) can be applied to predict SST variations due to heat fluxes across the air–sea interface. Here, the observed daily bandpass-filtered $Q_{\text {net}}$ anomalies are used as forcing to predict the resulting SST anomalies on S2S timescales at each grid point of the OAFlux dataset.

In order to determine the unknown model parameters $H_\text {m}$ and $\lambda$ that best describe the observed SST variability, the model in (11) was fit to the bandpass-filtered OISSTv2 anomalies. Alternatively, $H_\text {m}$ could have been treated as a forcing variable, e.g., by using data from reanalyses or climatologies (e.g., Deser et al. 2003). By treating the mixed layer depth as a model parameter, the predicted SST variability can be directly attributed to the heat fluxes.

Prior to the model fitting, the SST observations were remapped to the OAFlux grid using a first-order conservative interpolation scheme and then bandpass-filtered using the same Butterworth filter mentioned in Sect. 2.2. Generally, the mixed layer depth can vary throughout the year, but here, $H_\text {m}$ is assumed constant because the focus is on the winter months and the model was fit for this season only. This assumption has been shown to be suitable for predictions of low frequency SST variations (Lagerloef et al. 1998). Sensitivity experiments with time-varying mixed layer depth $H_\text {m}$ did not yield a significant improvement of the model fit to the observations.

The fitting procedure is based on an optimization algorithm that minimizes the mean squared error (e.g., Wilks 2011)

12 $$\begin{aligned} \text {MSE} = \frac{1}{N_{W}} \sum _{t\,\in \,W} \left( y_{t} - o_{t} \right) ^2 \end{aligned}$$

during winter, where $y_{t}$ and $o_{t}$ are the predicted and observed SST anomalies, respectively, and *W* is the set of $N_{W}$ daily time indices for each winter from 1985 to 2009. This minimization technique corresponds to maximum likelihood estimation under the assumption that the errors have a Gaussian distribution.

The parameter estimation was conducted independently for all OAFlux grid points in the North Atlantic where both the interpolated SST and $Q_{\text {net}}$ anomalies are available. To ensure physically interpretable results, the parameters were constrained as follows: 1 m $\le H_\text {m} \le$ 4000 m and $\lambda>$ 0 day^−1^.

For each grid point, SST anomalies were predicted using (11) with the optimized model parameters for the whole period 1985–2009 starting from an initial value of zero. The agreement between the observed and predicted winter SST anomalies was assessed using

13 $$\begin{aligned} R^{2} = 1 - \frac{\sum _{t\,\in \,W} \left( y_{t} - o_{t} \right) ^2}{\sum _{t\,\in \,W} (y_{t} - \overline{y})^{2}}, \end{aligned}$$

where $R^2 = 0$ and $R^2 = 1$ mean no and perfect agreement, respectively. This measure of fit is motivated by the coefficient of determination (e.g., Wilks 2011) used to assess the fit of multiple linear regression models. In the present case, the predictions by the mixed layer model (11) do not vary linearly with the two model parameters and so it is possible for $R^2$ to become negative.

In order to test if $R^{2}$ is significantly different from zero, a bootstrapping method was applied. The predicted time series was divided into blocks of 90 days for each winter from 1 December through 28 February. These blocks were randomly shuffled to create a synthetic time series which was then compared to the observed winter SST anomalies using  (13). This process was repeated 1000 times and from the resulting $R^{2}$ values, the quantiles of the underlying probability distribution were estimated. The $R^{2}$ of the original prediction is considered statistically different from zero at the 5% significance level if it is above the 95th percentile of the bootstrap distribution.

Figure 4a shows the model fit at the grid points in the study domain where $R^{2}$ is greater than the significance threshold. Generally, $R^{2}$ varies between 0.03 and 0.67 over large parts of the study domain. The best agreement of the predictions with the observations can be found in regions where the composite analysis in Sect. 3 showed a significant relationship between the MJO and SST. That is, maximum values of $R^{2}$ occur along the coast in the Gulf of Mexico. Similarly good model fit can be found on the continental shelf along the eastern seaboard of North America (particularly in the Middle Atlantic Bight), in the Sargasso Sea, and southwest of the Azores.

There are some regions where there is no significant agreement between the model and the observations. However, they correspond to areas without a significant relationship between the MJO and SST. In the Gulf Stream Extension Region, advection by large-scale currents and stirring by mesoscale variability play an important role for temperature changes in the surface mixed layer. Furthermore, SST in the subpolar gyre is strongly influenced by convective mixing. None of these oceanographic processes are captured in the mixed layer model and therefore, the poor model fit is expected in these regions. Overall, the significant values of $R^{2}$ demonstrate where net heat flux is important for determining the SST variability on S2S timescales in the North Atlantic.

As shown in Fig. 4b, the estimated mixed layer depth $H_\text {m}$ varies between 50 and 150 m over most parts of the study domain where the model fit is significant. Along the coasts of North Africa and the Gulf of Mexico, lower values can be identified. The estimated $H_\text {m}$ and its spatial distribution are in general qualitative agreement with available climatologies for the winter months (e.g., Kara et al. 2003; de Boyer Montégut et al. 2004).

Figure 4c shows the estimated damping rate $\lambda$. Over large areas of the North Atlantic $\lambda ^{-1} =$ 25–100 days and shorter relaxation timescales occur in the eastern Gulf of Mexico, in the South Atlantic Bight, and in the Bay of Biscay. These decay timescales for SST anomalies in response to forcing by air–sea heat fluxes are shorter than previously documented values of 3–6 months (e.g., Frankignoul 1985; Deser and Timlin 1997; Deser et al. 2003). This discrepancy is likely related to the bandpass-filtering of the net heat flux which excludes variations on timescales longer than 100 days.

Overall, the simple one-dimensional surface mixed layer model forced by net heat flux anomalies is able to capture a significant part of the observed SST variability in the MJO frequency band in the study region. This demonstrates that air–sea heat flux variations over the North Atlantic are a major driver of the observed winter SST variability on S2S timescales.

**Fig. 5:**
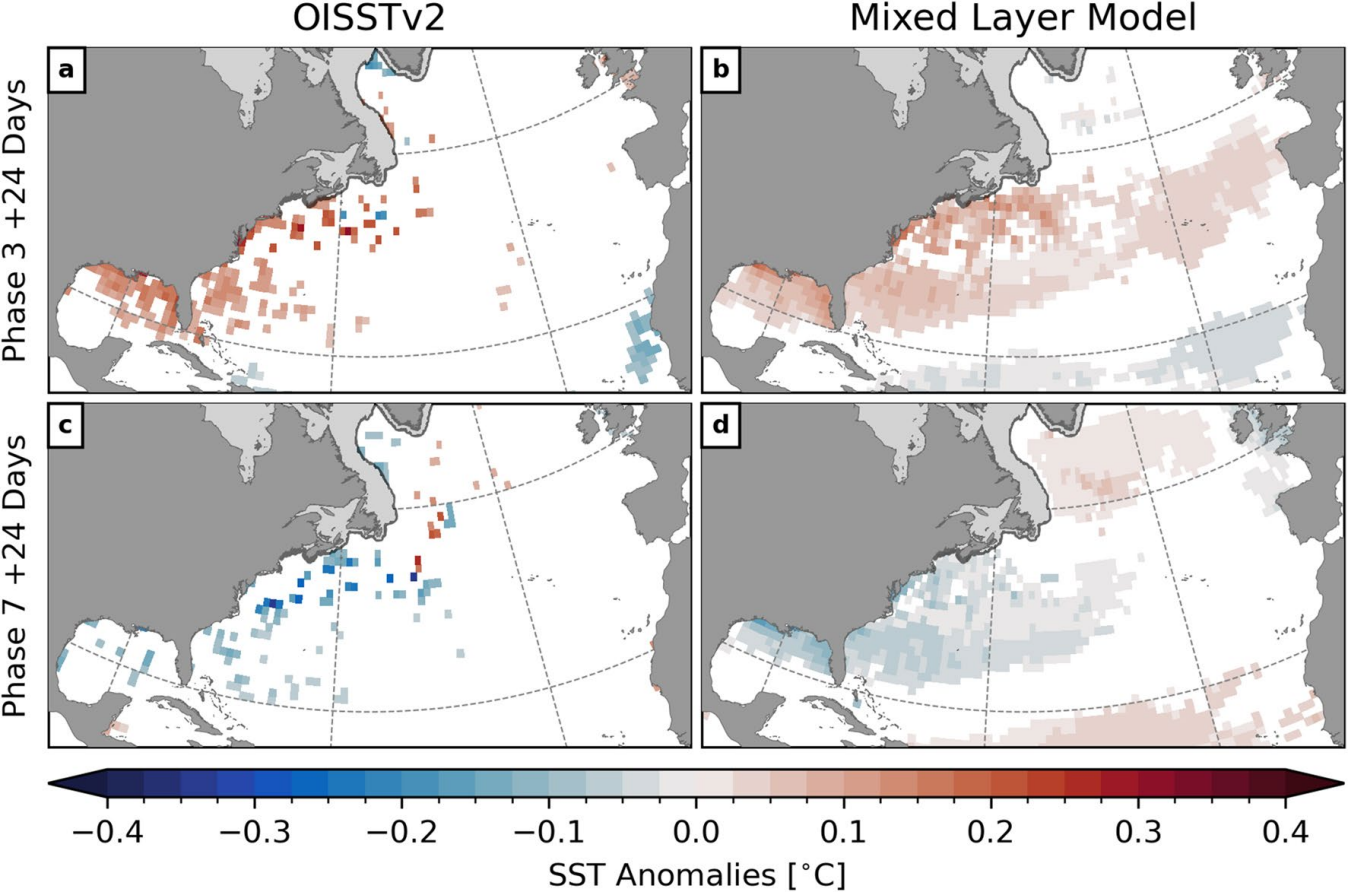
Composites of observed and predicted SST anomalies at lag $\delta =$ 24 days after phases 3 (**a**, **b**) and 7 (**c**, **d**) during winter when the RMM amplitude $A_\text {RMM}>$ 1. Only anomalies statistically different from zero at the 10% significance level are shown. Shaded areas near the coast show the climatological sea ice cover. Note that the composites of the observations were computed for the period 1981–2019

### Composites of SST predictions with respect to the MJO

The predictions by the mixed layer model are now used to test if the simple dynamics in that model can explain the observed SST signal with respect to the MJO. The focus will be on the predicted SST anomalies with respect to phases 3 and 7 which have been shown to initiate robust teleconnections in the northern hemisphere.

Using the same procedure described in Sect. 3, composites of SST anomalies predicted by the mixed layer model were computed with respect to the MJO. Figure 5 shows composites of observed and predicted SST anomalies at lag $\delta =$ 24 days after phases 3 and 7 during winter when the RMM amplitude $A_\text {RMM}>$ 1.

Following MJO phase 3, the composite of the SST predictions shows a large-scale positive temperature anomaly in the northern Gulf of Mexico and spanning the entire mid-latitude North Atlantic. The largest anomalies generally occur near the coast. Additionally, a weak cold anomaly can be seen across the northern tropical Atlantic.

At lag $\delta =$ 24 days after phase 7, the predicted SST composite shows a negative anomaly in the northern Gulf of Mexico and in the western North Atlantic. To the north and south of it, widespread warm anomalies are visible in the subpolar gyre and toward the equator. This is similar to the well known SST tripole pattern which has been associated with anomalies in sensible and latent heat flux on monthly to seasonal timescales (e.g., Cayan 1992b).

The spatial structure of the predicted and observed SST composites are qualitatively similar, but the predicted anomalies are generally smaller and extend further east across the North Atlantic. One possible reason is the mixed layer model predicts an average temperature over the surface mixed layer while the observations in OISSTv2 are corrected to represent a “bulk” SST at about 0.5 m depth (Reynolds et al. 1981). Another reason is that all parameters of the mixed layer model have been kept constant through time.

From the observed composites, it can be seen that the strongest SST anomalies occur in shallow regions near the coast. Due to the relatively coarse spatial resolution of the OAFlux dataset (1 ${}^{\circ }$ grid spacing) the predicted SST anomalies are representative of a larger area which, on the continental shelves, can cover water depths ranging from 10 to 100 m. This can lead to an underestimation of the SST anomalies.

Additionally, the predicted SST signal is generally smoother, and spatially more coherent, than the observed composites. This could be in part due to the different horizontal resolutions of OISSTv2 and OAFlux. More importantly, it suggests that processes which are not captured in the mixed layer model, e.g., advection, also contribute to the response of SST to the MJO.

The agreement of the sign and overall spatial structure of the observed and predicted composites supports the hypothesis that he large-scale SST change on S2S-timescales are driven primarily by anomalous air–sea heat fluxes caused by atmospheric perturbations linked to the MJO.

**Fig. 6:**
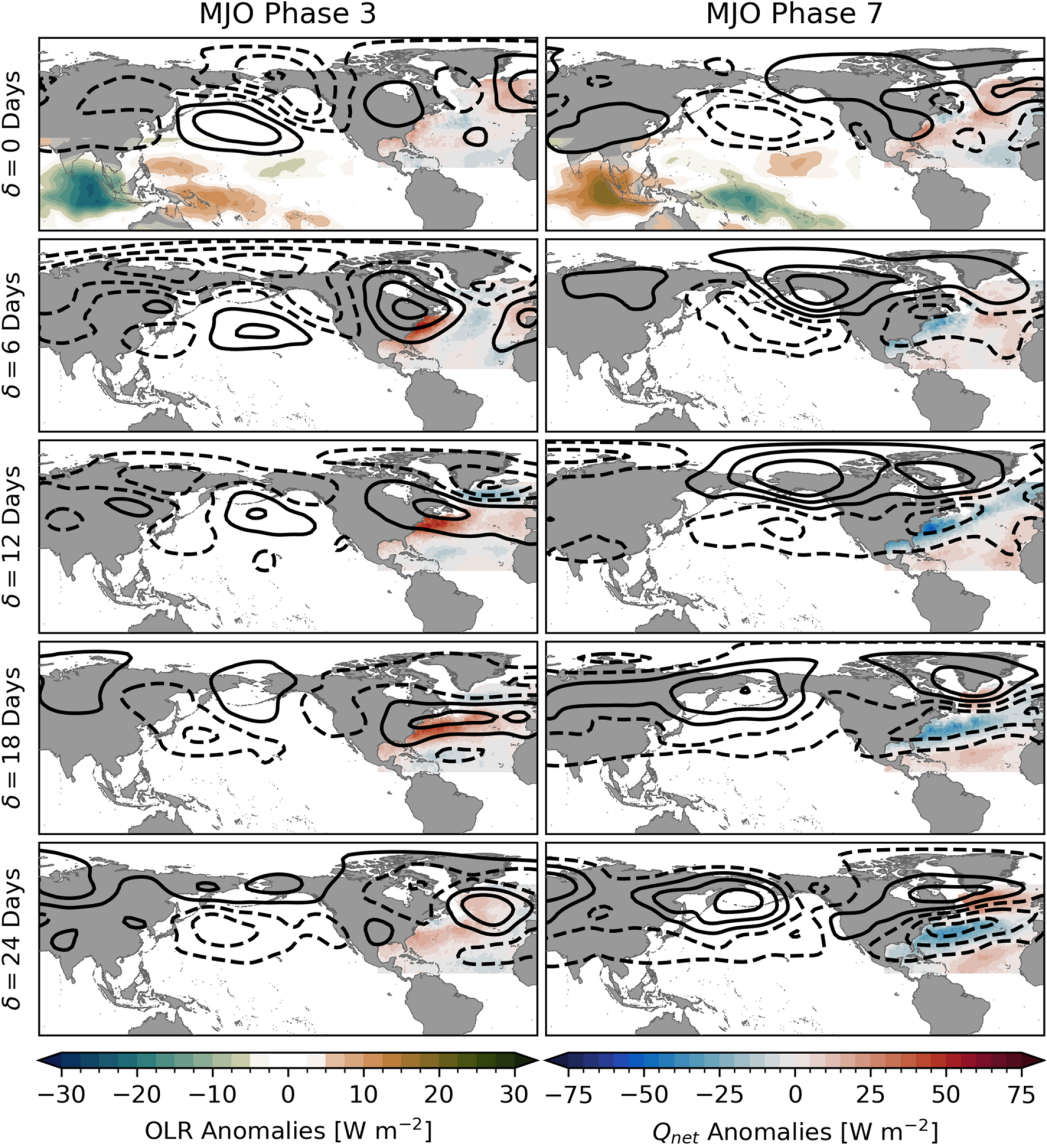
Atmospheric circulation anomalies during and after MJO phases 3 (left) and 7 (right). Bandpass-filtered outgoing longwave radiation (OLR, left color scale) anomalies are only shown at lag $\delta =$ 0 days over the Indian Ocean, Maritime Continent, and western Pacific to illustrate the large-scale convection anomaly associated with the MJO. Negative (positive) values refer enhanced (suppressed) convection. Contours show anomalies of 500 hPa geopotential height (interval 20 m, zero line omitted) illustrating the Rossby wave train that propagates from the tropics to the North Atlantic region in response to the MJO. Dashed lines refer to negative values. In the North Atlantic, composites of net heat flux ($Q_{\text {net}}$, right color scale) are shown. These fields are a subset of the composites shown in Figure A1

### Physical mechanisms and teleconnections

In order to link the heat flux composites to the MJO, we examined composites of atmospheric circulation anomalies during and after MJO phases 3 (Fig. 6, left column) and 7 (Fig. 6, right column). Rows correspond to lags $\delta =$ 0, 6,..., 24 days after these phases. The top panels also show composites of daily bandpass-filtered NOAA interpolated outgoing longwave radiation (OLR, Liebmann and Smith 1996, obtained from the NOAA Physical Sciences Laboratory) anomalies over the Indian Ocean, Maritime Continent, and western Pacific which is a proxy for the large-scale atmospheric convection associated with the MJO. Phase 3 is characterized by a dipole of enhanced convection over the eastern Indian Ocean and suppressed convection over the western tropical Pacific. Half a MJO cycle later, during phase 7, the sign of this dipole is reversed.

Composites of 500 hPa daily geopotential height anomalies from the NCEP-DOE AMIP-II Reanalysis (Kanamitsu et al. 2002) are shown as contours with dashed lines indicating negative values. In the North Atlantic, composites of net heat flux anomalies $Q_{\text {net}}$ are shown (see also Appendix).

The series of alternating positive and negative anomalies of geopotential height illustrate the Rossby wave train that is induced by anomalous diabatic heating and cooling associated with the MJO convective dipole (e.g., Sardeshmukh and Hoskins 1988) and has been shown to influence the extratropical atmospheric circulation (e.g., Lin and Brunet 2009; Lin et al. 2010; Seo et al. 2016). It has been shown that this pattern leads to an anomalous northward geostrophic advection of warm and moist air over the eastern United States after MJO phase 3 causing a significant increase in surface air temperature over all of Northeast America (Seo et al. 2016). As illustrated in Fig. 6, the resulting warm anomaly leads to an increased net air–sea heat flux into the surface ocean along the eastern seaboard of North America.

During and after MJO phase 7, the large-scale atmospheric circulation is, to first order, a mirror image of the response to phase 3 with reversed sign (right column in Fig. 6). This results in a southward flow over North America and the western North Atlantic advecting cold and dry air masses from polar regions. Consequently, a negative heat flux anomaly occurs along the eastern seaboard and in the northern Gulf of Mexico at lag $\delta =$ 6 days. The positive heat flux anomaly in the eastern North Atlantic at the same time can be explained by advection of warm and humid air from the south in agreement with the shown atmospheric circulation.

With increasing lag after both phase 3 and 7, the geopotential height anomalies are reminiscent of the North Atlantic Oscillation (NAO) in agreement with previous studies (e.g., Cassou 2008; Lin et al. 2009). This also includes the resulting pattern of net heat fluxes related to the NAO on monthly to seasonal timescales that can be largely explained by changes in sensible and latent heat fluxes (e.g., Cayan 1992a, b; Visbeck et al. 2003; Somavilla Cabrillo et al. 2011).

Overall, the atmospheric circulation in response to the MJO can explain the spatial structure of the net heat flux composites in the extratropical North Atlantic. This provides a physical mechanism for the teleconnection between the MJO and SST composites shown in Fig. 3. Furthermore, it explains why the strongest SST response to the MJO occurs along the eastern seaboard of North America. This region is influenced by changes in the atmospheric circulation due to the Rossby wave train only a few days after it is induced in the tropics. As the atmospheric response spreads and intensifies across the North Atlantic, the heating or cooling of the surface ocean continues and therefore, a strong SST anomaly develops.

This raises the following questions: (1) what is the ocean response to the MJO at smaller spatial scales? (2) How is the effect of atmospheric forcing in response to the MJO vertically projected into the subsurface ocean? and (3) how do other physical processes (e.g., advection and coastal upwelling) contribute to the mean ocean response to the MJO? In the following section we address these question using a high-resolution regional model of the Gulf of Maine and Scotian Shelf region. This will also allow us to test the feasibility of using such models for ocean prediction on S2S timescales.

## High-resolution model of the ocean response on the shelf

In order to analyze the ocean response to the MJO at smaller spatial scales and at depth, we use a numerical ocean circulation model of the Gulf of Maine and Scotian Shelf, henceforth referred to as GoMSS. In contrast to the mixed layer model, GoMSS is a high-resolution regional model capturing all relevant physical processes that influence the ocean and shelf circulation, e.g., advection by large-scale currents, tidal mixing, and stirring by mesoscale variability. Therefore, it is a suitable tool to further explore the ocean response related to the MJO and its interaction with other ocean processes.

### Gulf of Maine and Scotian Shelf (GoMSS) ocean model

**Fig. 7:**
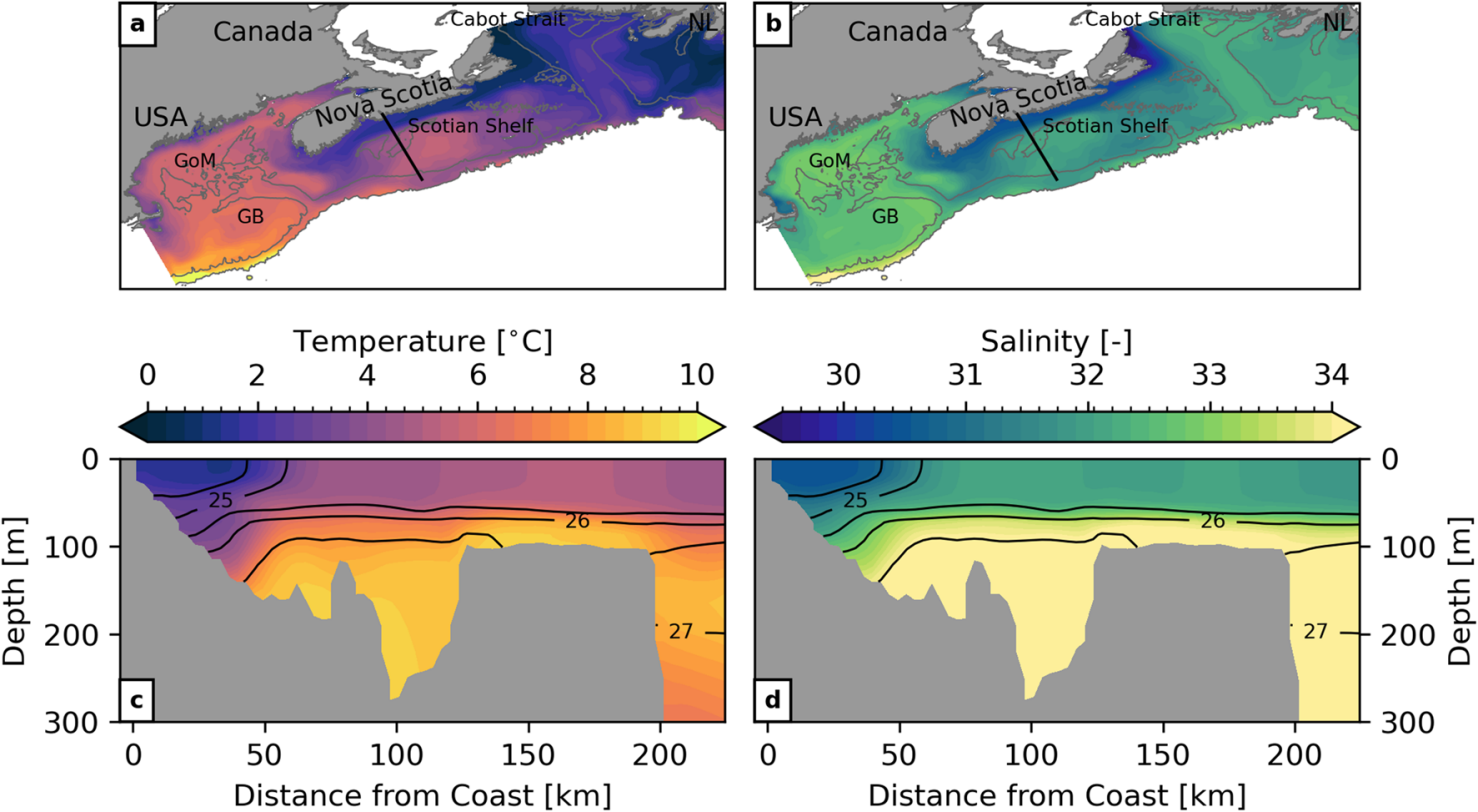
Mean hydrographic conditions predicted predicted by the GoMSS control run P0. **a** Mean sea surface temperature. **b** Mean surface salinity. The maps show model grid points where the water depth <2000 m. **c** Ocean temperature and **d** salinity along the Scotian Shelf section marked by the black line in panels a and c. Black contours show the associated in-situ density $\sigma = \rho -$1000 kg m$^{-3}$. Abbreviated locations referenced in the text: Gulf of Maine (GoM), Grand Banks (GB), Newfoundland (NL)

GoMSS was initially developed by Katavouta and Thompson (2016) based on the Nucleus for European Modelling of the Ocean (NEMO; Madec et al. 2017). The model domain covers the continental shelf from the western Grand Banks to Cape Cod including the Gulf of Maine and Bay of Fundy as well as adjacent parts of the Atlantic Ocean (see Fig. 7).

Here we use an upgraded configuration based on version 3.6 of NEMO. GoMSS has a horizontal grid spacing of 1/36 ${}^{\circ }$ which corresponds to 2.1–3.6 km in the study region. The vertical model grid consists of 52 levels which, in a state of rest, increase in thickness from 0.72 m at the surface to 235.33 m at the bottom. The maximum depth of the bathymetry is clipped at 4000 m. GoMSS uses a variable volume formulation of the nonlinear free surface (z*-coordinates) which means the thickness of all model levels varies over time, scaled by sea surface height (Levier et al. 2007). At the bottom, partial cells are applied to better resolve the bathymetry.

**Table 1:** Overview of boundary forcing for model experiments with GoMSS

	Spin-up period				Analysis period			
	1 October–31 December				1 January–18 February			
Run	$\overline{F}$	$F'_\text {a}$	$\tilde{F}_\text {o}$	$\hat{F}$	$\overline{F}$	$F'_\text {a}$	$\tilde{F}_\text {o}$	$\hat{F}$
P0	$\checkmark$	$\checkmark$	$\checkmark$		$\checkmark$	$\checkmark$	$\checkmark$	
P3	$\checkmark$	$\checkmark$	$\checkmark$		$\checkmark$	$\checkmark$	$\checkmark$	$\hat{F}(3, \delta )$
P7	$\checkmark$	$\checkmark$	$\checkmark$		$\checkmark$	$\checkmark$	$\checkmark$	$\hat{F}(7, \delta )$

Each column refers to atmospheric and ocean forcing in a frequency band defined in (14). The checkmarks indicate which forcing frequencies are applied. See text for details

### Specification of boundary forcing and model experiments

Along the open boundaries, GoMSS is forced with daily output from the Global Ocean Physical Reanalysis product (GLORYS12v1, 1/12 ${}^{\circ }$ grid spacing, Lellouche et al. 2021). Sea level, currents, ocean temperature, and salinity are prescribed using the Flow Relaxation Scheme (Davies 1976; Engedahl 1995) which smoothly introduces the external data over 10 grid cells adjacent to the open boundaries. Additionally, tidal elevation and depth-averaged currents for five constituents (M_2_, N_2_, S_2_, K_1_, O_1_) from the barotropic Finite Element Solution global tide model (FES2004; Lyard et al. 2006) are prescribed using the Flather radiation scheme (Flather 1994). No nudging or data assimilation is applied in the interior of the model domain.

At the air–sea interface, GoMSS is forced with hourly data from the NCEP Climate Forecast System Reanalysis (CFSR; Saha et al. 2010). The atmospheric forcing is calculated in GoMSS using the Coordinated Ocean-Ice Reference Experiment (CORE) bulk formulae (Large and Yeager 2004) for air–sea fluxes of heat and momentum.

For this study, we specify an idealized model forcing *F* along the open boundaries and at the air–sea interface based on GLORYS12v1 and CFSR, respectively. This forcing is constructed based on contributions from four frequency bands that we consider to be the dominant timescales at play:

14 $$\begin{aligned} F = \overline{F} + F'_\text {a} + \tilde{F}_\text {o} + \hat{F}(j, \delta ), \end{aligned}$$

where $\overline{F}$ is the seasonal climatology, $F'_\text {a}$ is high-frequency atmospheric variability with timescales of 10 days or less, $\tilde{F}_\text {o}$ are tidal variations (only applied at the lateral ocean boundaries), and $\hat{F}(j, \delta )$ is the forcing on S2S timescales (15–100 days) depending on the lag $\delta$ after a given MJO phase $\Phi _\text {RMM} = j$. Using this decomposition, it is possible to identify the effect of the different forcing timescales on the circulation and hydrography in the GoMSS domain. In order to isolate the predicted mean ocean response to the MJO, model sensitivity runs with and without $\hat{F}$ are performed.

It is important to recognize $\overline{F}$ describes a smoothly varying seasonal mean of the atmosphere-ocean system. (Due to the higher heat capacity of water, the surface ocean does not fully adjust to the cooling by the atmosphere during winter.) Therefore, it is important to include the seasonal cycle in $\overline{F}$ as opposed to a seasonal mean value which would lead to an overestimation of the winter cooling. The daily climatology of the forcing variables was computed for the period 1993–2010 using the methodology described in Sect. 2.2. The forcing on synoptic and S2S timescales is extracted by applying filters to the anomalies from this climatology.

Stochastic, high-frequency variability in the atmospheric forcing $F'_\text {a}$ is necessary for a realistic prediction of the hydrography, particularly the mixed layer depth. $F'_\text {a}$ includes weather events on synoptic timescales, e.g., storms passing through the study area. In order to compute $F'_\text {a}$, a third order Butterworth highpass-filter (Butterworth 1930) with a 10-day cutoff was applied to the anomalies at each grid point of the atmospheric forcing. The filtered anomalies for the period 1 October 1992 to 18 February 1993 were then used to define the high-frequency atmospheric forcing $F'_\text {a}$. This period was chosen based on the reduced activity of the MJO (mean $A_\text {RMM}$ = 1.2). Given the known connection between the MJO and the El-Niño Southern Oscillation (ENSO, e.g., Lee et al. 2019) the period for the high-frequency forcing was also chosen to be a during a neutral year indicated by the Oceanic Niño Index of 0.1 ${}^{\circ }\text {C}$ during DJF 1992/93. Sensitivity studies using anomalies from a different year showed that all results presented below are robust to changes in the period of the high-frequency forcing.

The atmospheric and ocean forcing on S2S timescales $\hat{F}$ is based on lagged composites with respect to the MJO which were calculated following the methodology outlined in Sect. 3. Prior to compositing, the variability of the forcing variables in the MJO frequency band was extracted by applying a third order Butterworth bandpass filter with a 15- to 100-day passband to the anomalies. Additionally, any remaining interannual variability was removed by subtracting the mean anomaly of each winter season. The time-varying forcing composites $\hat{F}(j, \delta )$ were then computed based on (7) depending on the MJO phase *j* and lag $\delta$ (see details below). The impact of $\hat{F}$ along the lateral boundaries is assumed to be smaller than the atmospheric forcing and will not be further discussed.

An alternative approach to the decomposition of *F* in (14) would be the specification of $\hat{F}$ by extracting the MJO-related forcing using a linear regression of the raw reanalysis fields onto the RMM index (e.g., Oliver 2015). The difference between a model experiment using the full reanalysis fields as forcing *F* and a run using $F - \hat{F}$ would allow the response to the MJO to be quantified. This approach can be useful for identifying the ocean response to a canonical cycle or individual episodes of the MJO. However, it is not straightforward to specify the known time lag between a specific MJO phase and the atmospheric and ocean response which can vary with location. For this reason, the composite-based approach is used in this study; it provides a prediction of the mean ocean response to the MJO and its lagged behaviour can be directly assessed with respect to a particular phase. It is also a natural extension of the composite analysis of the SST observations in Sect. 3.

Three model experiments were performed to downscale the mean ocean response with focus on MJO phases 3 and 7 during winter. Each model run was for the period 1 October to 18 February with the first three months discarded to avoid contamination by model spin-up. The response to each phase is predicted in a separate model run (P3 and P7) which is then compared to a control run (P0) without $\hat{F}$ (see Table 1). All model runs were initialized with the climatology for 1 October and spun up over three months until 31 December using the neutral forcing

15 $$\begin{aligned} F_\text {n} = \overline{F} + F'_\text {a} + \tilde{F}_\text {o}. \end{aligned}$$

The control run P0 is based on continued forcing with $F_\text {n}$, whereas for P3 and P7, the composites $\hat{F}(j, \delta )$ were added for $j =$ 3 and $j =$ 7, respectively. It is assumed that the MJO occurs in a given phase on 1 January and thus the contemporaneous composites ($\delta =$ 0) were included in the forcing for that day. This added forcing is small enough that no additional spin-up was necessary. For the subsequent days, composites for increasing lags $\delta>$ 0 were added. Each model run was continued until 18 February which is equivalent to a lag $\delta$ = 48 days after a given phase and corresponds to the mean period of the MJO. The downscaled mean ocean response to forcing in the MJO frequency band is then obtained by subtracting the control run P0 from P3 and P7, respectively. In the following, only the 49-day, post spin-up period will be discussed.

Figure 7 shows the mean sea surface temperature and salinity predicted by the control run P0 for the analysis period focusing on the model grid points with water depth <2000 m. In the study region, colder, less salty waters can be seen off the coast of Newfoundland (NL) and warmer, slightly more saline waters in the Gulf of Maine (GoM) and on Georges Bank (GB). Cold and relatively fresh water enters the model domain through the western Cabot Strait and follows the Nova Scotia Current along the coast toward the Gulf of Maine. The outer edge of the current can be identified by the strong gradients of temperature and salinity confining this water mass to the nearshore on the Scotian Shelf.

This separation is also visible in mean hydrographic conditions along the section across the Scotian Shelf marked by the black line and shown in the bottom panels of Fig. 7. Henceforth, this will be referred to as the Scotian Shelf section. The horizontal density gradient in the surface mixed layer marks the front separating the colder and fresher water mass associated with the Nova Scotia Current from the rest of the shelf. Across the entire Shelf, the water column is stratified with colder, fresher water in the surface mixed layer with depth of about 60–80 m. This is in close qualitative agreement with the winter climatology computed from glider-based observations by Dever et al. (2016). The realism of the seasonal mean state predicted by the control run P0 is further supported by its similarity to the results of Katavouta and Thompson (2016) and Katavouta et al. (2016) who demonstrated that GoMSS improves the representation of shelf circulation compared to HYCOM, a global system with lower resolution. They furthermore showed that GoMSS provides realistic predictions of the tidal variability in the region as well as their dynamical interaction on tidal and seasonal timescales.

**Fig. 8:**
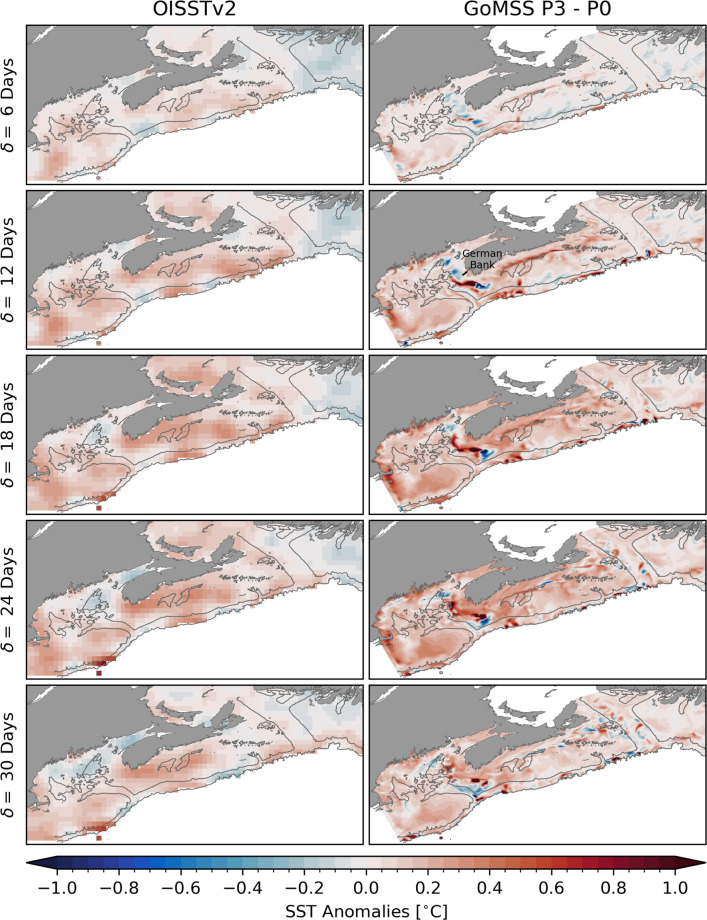
Observed composites and predictions of SST anomalies with respect to MJO phase 3. Left column: composite maps of bandpass-filtered, observed SST anomalies when $A_\text {RMM}>$ 1 during winter calculated for the period 1993–2010. Right column: SST anomalies predicted by GoMSS (P3–P0). All anomalies are relative to lag $\delta =$ 0 days

**Fig. 9:**
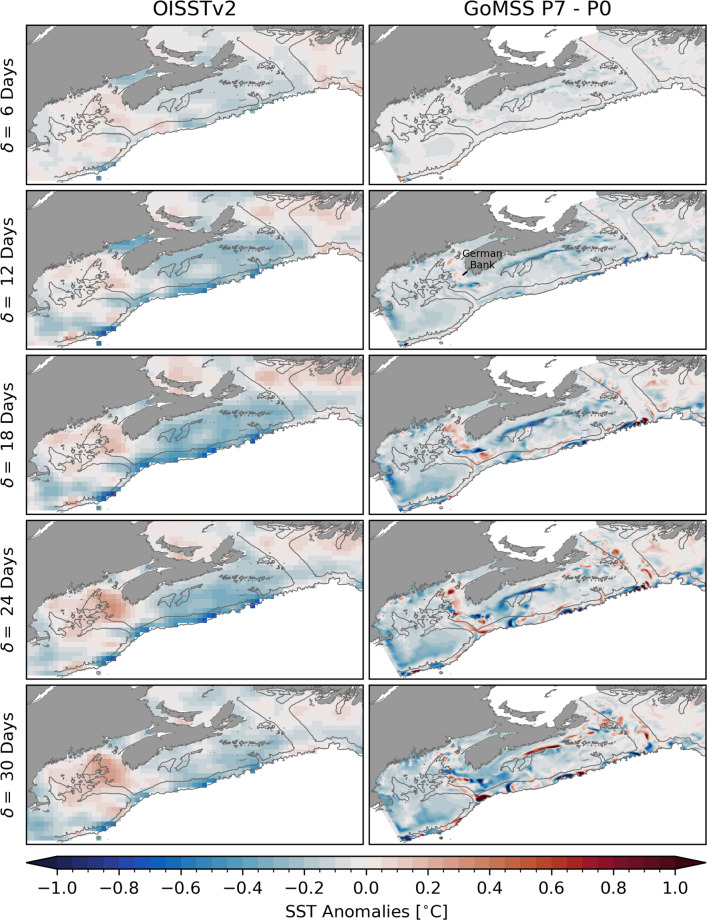
Observed composites and predictions of SST anomalies with respect to MJO phase 7. The format is the same as Fig. 8

### Predicting the observed composites of SST

The ocean response in the Gulf of Maine and Scotian Shelf to the MJO-related forcing is analyzed by comparing the deviations of the GoMSS model runs P3 and P7 from the control run P0. These differences will henceforth be referred to as anomalies.

The spatial structure of the surface ocean response to MJO phase 3 in the Northwest Atlantic shelf region is shown in Fig. 8. In the left column, composite maps of bandpass-filtered, observed SST anomalies relative to zero lag are shown for $\delta =$ 6, 12, ..., 30 days. These composites have been computed as described in Sect. 3 for the same period as the model forcing (1993–2010). To make the observed composites more comparable to the results of the sensitivity experiments with GoMSS, anomalies at lag $\delta =$ 0 days have been subtracted. As illustrated in Sect. 3, a wide-spread warm anomaly occurs in the study region 2 to 3 weeks after the MJO is in phase 3.

The panels in the right column of Fig. 8 show SST anomalies predicted by GoMSS in response to the composite forcing calculated as the difference P3−P0. It can be seen that the forcing $\hat{F}$ leads to a large-scale surface warming in the study area reaching its maximum 18–24 days after the MJO is in phase 3. This predicted increase in SST is broadly consistent, for low wavenumber spatial patterns, with the observed composites. The strongest response is found on Georges Bank and on the Scotian Shelf. Generally, the maximum surface warming predicted by GoMSS is slightly stronger and contains small-scale features that cannot be captured in the gridded observations due to their coarser resolution.

Similarly, GoMSS is able to capture the large-scale cooling trend after MJO phase 7 which is evident in the observed composites in the left column of Fig. 9. The strongest decrease in SST is observed on the eastern Scotian Shelf at lags $\delta =$ 18–24 days. In the panels on the right of Fig. 9, the predicted SST anomalies (P7–P0) are shown for the period after MJO phase 7. GoMSS slightly underestimates this cooling and, contrary to the observations, predicts a stronger response on the western Scotian Shelf to occur later at $\delta$ = 24–30 days. Furthermore, the model predicts an overall surface cooling throughout the Gulf of Maine where the observations suggest a small positive anomaly that does not occur when the full period of the OISSTv2 dataset is used (not shown).

The predicted SST response is consistent with forcing due to the large-scale atmospheric anomalies following MJO phases 3 and 7 shown in Fig. 6. This furthermore is consistent with and complements the results of the simplified surface mixed layer model in Sect. 4.

Due to its higher horizontal resolution, GoMSS is also able to predict localized features, e.g., a narrow band of large anomalies along the outer edge of the Nova Scotia Coastal Current and off the coast of southwest Nova Scotia. In these areas, strong SST gradients exists (see Fig. 7). Due to the MJO-related forcing, these gradients are shifted horizontally leading to anomalies of >1 ${}^{\circ }\text {C}$. This is further discussed below in Sect. 5.4.

The observed SST composites are only partly comparable to the model predictions because the GoMSS runs are based on idealized, composited forcing. Nevertheless, the qualitative agreement between the predicted large-scale anomalies and the results in Sect. 4 provides further confidence that the ocean response is driven by atmospheric perturbations caused by the MJO.

**Fig. 10:**
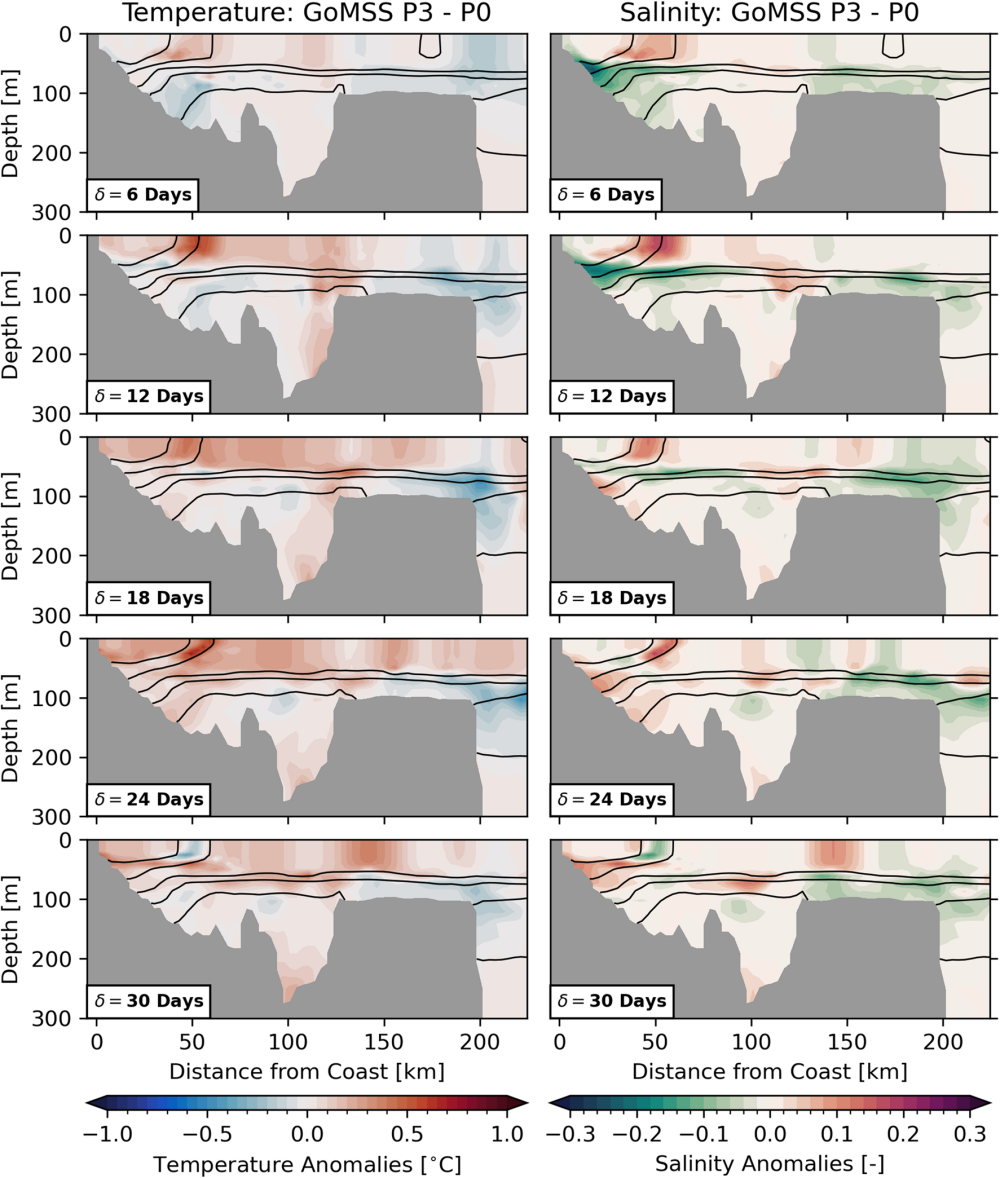
Ocean temperature and salinity anomalies along the Scotian Shelf section with respect to MJO phase 3 predicted by GoMSS (P3–P0). Contours show the predicted in-situ density $\sigma$

**Fig. 11:**
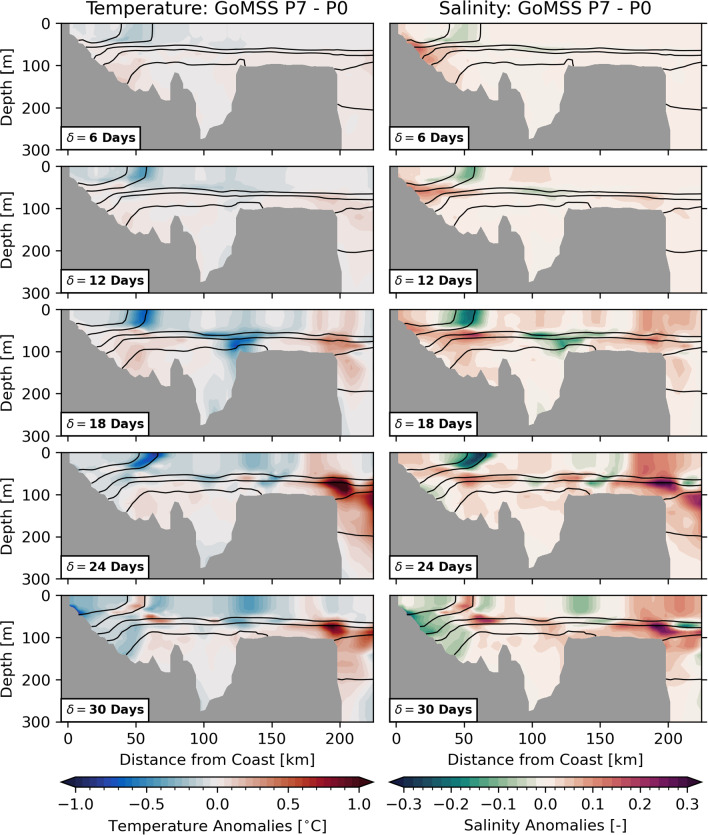
Ocean temperature and salinity anomalies along the Scotian Shelf section with respect to MJO phase 7 predicted by GoMSS (P7–P0). Contours show the predicted in-situ density $\sigma$

**Fig. 12:**
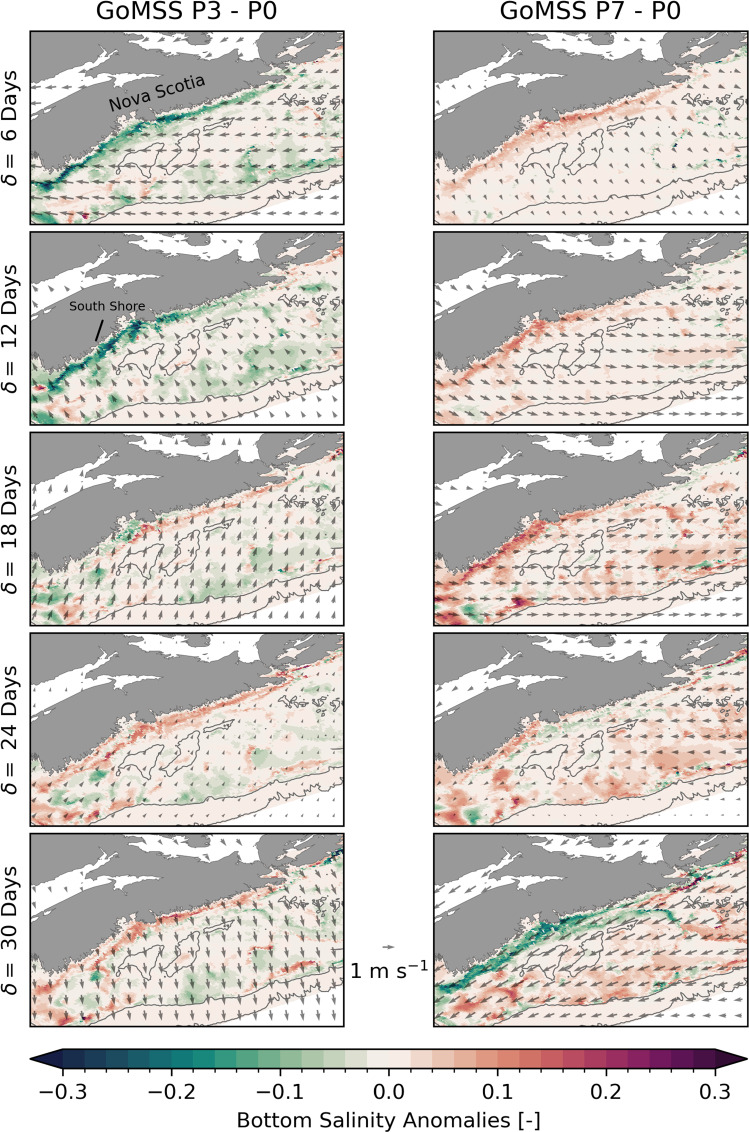
Bottom salinity anomalies predicted by GoMSS (left column: P3–P0, right column: P7–P0). Vectors show the composites of bandpass-filtered CFSR wind anomalies at 10 m height with respect to MJO phases 3 (left column) and 7 (right column) when $A_\text {RMM}>$ 1 during winter. Note that the Bay of Fundy region has been masked out

### Predicted subsurface and small-scale ocean variability

How deep does the signal at the surface penetrate into the subsurface ocean? Figures 10 and 11 show the ocean response to MJO phases 3 and 7, respectively, predicted by GoMSS along the Scotian Shelf section. The warm anomaly with its maximum at $\delta =$ 18–24 days after phase 3 extends from the coast beyond the shelf break, but is generally confined to the surface mixed layer. As noted in Sect. 5.3, the wide-spread cold anomaly in response to phase 7 is predicted to occur mainly on the western Scotian Shelf at longer lags. Therefore, this signal is more dominant along the section at $\delta =$ 30 days, but also does not penetrate beyond the pycnocline.

After both MJO phases 3 and 7, a strong and localized temperature anomaly occurs along the density front at the outer edge of the Nova Scotia Current and in the eastern Gulf of Maine at lags $\delta =$ 12–24 days. These correspond to the narrow anomaly parallel to the coastline seen in Figs. 8 and 9. Similar anomalies can be seen in the salinity along the Scotian Shelf section. This suggests a horizontal displacement of the density front due to advection toward and away from the coast after phase 3 and 7, respectively. The horizontal resolution of OISSTv2 (0.25 ${}^{\circ }$ grid spacing) is too coarse to resolve these features.

In regions where the surface mixed layer does not extend throughout the water column, bottom salinity can act as a tracer for the ocean circulation at depth. Figure 12 shows the bottom salinity anomalies on the Scotian Shelf following MJO phases 3 and 7 predicted by GoMSS. Vectors show the composites of bandpass-filtered 10 m wind anomalies that are part of the model forcing $\hat{F}$. After phase 3, the wind anomalies are directed predominantly in the onshore direction. The anomalous wind forcing causes the density front of the Nova Scotia Current to move toward the coast where anomalous downwelling of colder and fresher surface water occurs. This results in a negative bottom salinity anomaly along the entire Atlantic coastline of mainland Nova Scotia, which becomes more pronounced along its South Shore at $\delta =$ 12 days. With increasing lag, the direction of the near-surface wind anomalies changes and a positive bottom salinity anomaly occurs.

The opposite effect can be seen after MJO phase 7 when the near-surface wind anomalies lead to upwelling-favourable conditions. As a result, the Nova Scotia Current widens and the density front along its outer edge is advected offshore (Fig. 9). Positive anomalies in bottom salinity at lags $\delta =$ 6–18 days indicate more saline water is upwelled into the surface layer along the coast.

The order of magnitude of the predicted upwelling-related salinity anomalies is consistent with an advective change $D\frac{\partial S}{\partial z}$ assuming a salinity gradient $\frac{\partial S}{\partial z} =$ $-$0.03 m$^{-1}$ and a vertical displacement over 10 days of $D =$ 10 m. The coastal upwelling and downwelling signatures are less apparent in the temperature profile along the Scotian Shelf section (Figs. 10, 11). This is likely because the temperature changes in the surface mixed layer are mitigated by anomalous heat fluxes.

With increasing lag after MJO phases 3 and 7, the direction of the wind anomalies reverses leading to anomalous downwelling and upwelling, respectively, at lag $\delta =$ 30 days. Given the mean period of a MJO cycle of 48 days, a lag of $\delta =$ 24 days after phase 3 roughly corresponds to the time when the MJO is in phase 7. Therefore, there is a similarity in the bottom salinity anomaly along the coast in the bottom left and top right panel of Fig. 12.

From Fig. 11, it is apparent that strong anomalous upwelling also occurs along the shelf break after phase 7 leading to intrusions of warm and salty waters onto the shelf. This is consistent with observations by Petrie (1983) who showed that this intense upwelling is the response to transient winds in alongshelf direction over a period of two days or longer. Similarly, downwelling occurs after phase 3, but the resulting anomalies are less pronounced (Fig. 10).

In the eastern Gulf of Maine, the localized SST anomalies shown in Figs. 8 and 9 are subject to the strong tidal flow in that region. This leads to enhanced vertical mixing over German Bank that causes the anomalies to extent throughout the whole water column beyond the wind-driven surface mixed layer (not shown).

Overall, it is apparent that the atmospheric perturbations caused by the MJO not only lead to a large-scale surface signal, but also affect the ocean circulation on smaller horizontal scales and the ocean interior. This demonstrates the benefits of a high-resolution ocean model like GoMSS which is able to better resolve the coastline and nearshore bathymetry, and also additional processes, e.g., tides.

## Summary and discussion

In this study, we examined the ocean response to the MJO in the northwest Atlantic and adjacent shelf seas using a variety of approaches ranging from simple, statistically based models to a regional, high-resolution ocean circulation model.

Based on composite analysis, a statistically significant relationship has been found between the MJO and large-scale changes of observed winter SST anomalies in the North Atlantic. A widespread positive anomaly develops in the Gulf of Mexico and in the western North Atlantic reaching its maximum at lags $\delta =$ 20–25 days after MJO phase 3 depending on the location. Due to the quasi-periodicity of the MJO, a similar anomaly pattern can also be observed after phases 4–7, but at shorter lags, illustrating the ambiguity as to which MJO phase initiates the teleconnection and the time lag at which it occurs (Fig. 3). The same issue arises after the other half of the canonical MJO cycle when a large-scale negative anomaly occurs in the same regions after MJO phases 7 and 8.

In general, the strongest SST anomalies occur in the northern Gulf of Mexico and in shallow, coastal regions along the eastern seaboard of North America, particularly in the Middle Atlantic Bight. Along the path of the Gulf Stream, the SST response to the MJO is masked by mesoscale eddy variability which increases the background noise level in that region. A significant part of this signal also extends offshore to the Sargasso Sea where the surface ocean variability is predominantly determined by local air–sea heat fluxes and convergence of Ekman heat transport (e.g., Buckley et al. 2014).

Clearly, the composite analysis cannot determine cause and effect. However, the large-scale structure of the SST anomalies suggests that they are driven by atmospheric perturbations in response to the MJO. This is supported by composites of net air–sea heat fluxes conditioned on the MJO which revealed anomalous heat exchange between the ocean and atmosphere prior to the SST anomalies. These heat fluxes arise from advection of warm and humid or cold and dry air masses which lead to anomalous sensible and latent heat exchange between the ocean and the atmosphere. Note that this study only focused on the response of the surface ocean to atmospheric perturbations related to the MJO. A possible feedback from the resulting SST anomalies to the atmosphere and potential downstream coupling with the tropics (e.g., Lin et al. 2009) is beyond the scope of this study.

Additionally, predictions by a simplified surface mixed layer model demonstrated that a significant part of the SST variability in the North Atlantic on S2S timescales is determined by net heat flux anomalies. Composites of the predicted SST anomalies showed a remarkable agreement with the observed relationship with the MJO. Overall, these results are consistent with Deser and Timlin (1997) who demonstrated a strong relationship between heat fluxes and SST tendencies in the North Atlantic on weekly timescales. The spatial structure of both the SST and heat flux composites can be related to other modes of climate variability in the northern hemisphere (e.g., Pacific-North American Pattern, PNA and NAO) that have been shown to be linked to the MJO through a Rossby wave train.

Note that the lag $\delta$ is the sum of the timescales for the Rossby wave train to be established between the tropics and the Atlantic Sector ($\delta _\text {T}$) and for the ocean to respond to anomalies in the atmospheric circulation ($\delta _\text {O}$). It is well established that $\delta _\text {T}$ is on the order of 1–2 weeks (e.g., Cassou 2008; Lin et al. 2009; Riddle et al. 2013). Similarly, it has been shown that there is a 2–3 week lag between large-scale atmospheric modes of variability and corresponding SST anomalies on weekly timescales Deser and Timlin (1997). This shows that the estimated $\delta$ from the composite analysis is plausible and furthermore helps determine which phase is more likely to initiate the teleconnection between the MJO and SST in the North Atlantic.

The mixed-layer model does not capture physical processes that can lead to an ocean response on smaller scales or in the subsurface (e.g., advection). Therefore, we examined the subsurface and small-scale ocean variability on S2S timescales using the regional model GoMSS to downscale the mean response to the MJO in the Gulf of Maine and Scotian Shelf region. Three model experiments were performed using idealized forcing decomposed into contributions from four frequency bands. To explicitly include the effect of the MJO, composites with respect to a specific phase were added to the time-varying neutral forcing. This neutral forcing consisted of contributions from the seasonal climatology, high-frequency atmospheric variability, and tidal variations in the ocean.

GoMSS predicts a large-scale positive SST anomaly following phase 3 and a widespread cooling of the sea surface after phase 7. In both cases, the maximum response occurs at lag $\delta =$ 18–24 days. Overall, the spatial structure, magnitude, and timing of the anomalies predicted by GoMSS are in agreement with the composite analysis of the SST observations with respect to the MJO. This shows GoMSS can reproduce the mean ocean response to the MJO. Consistent with the the predictions by the simplified mixed layer, the large-scale anomalies throughout the whole model domain were shown to be directly related to surface forcing on S2S timescales.

Additionally, the predictions by GoMSS reveal signals that cannot be identified from the observations. Horizontal advection in response to the anomalous wind forcing related to the MJO displaces the density front that separates the Nova Scotia Current from the rest of the shelf. This is expressed by a narrow band of strong SST anomalies parallel to the coast which is neither captured in the observations nor in the mixed layer model.

Predicted temperature profiles along the Scotian Shelf section showed the surface warming and cooling in response to the MJO generally do not extend beyond the mixed layer. However, in shallow areas where strong tidal mixing occurs, the signal can penetrate to the seafloor. This can have biological impacts as groundfish, lobster and other benthic species as well as their habitats have to adjust to these changes in temperature (e.g., Crossin et al. 1998; Donaldson et al. 2008; Burdett et al. 2019).

The anomalous wind forcing in response to the MJO leads to anomalous downwelling and upwelling along the coast of Nova Scotia and at the shelf edge after phases 3 and 7, respectively. If the intrusion of nutrient-rich slope water occurs toward the end of the winter, this could potentially contribute to a more pronounced spring bloom of phytoplankton on the Scotian Shelf. It is therefore possible that the MJO can also have an effect on biological processes.

Overall, based on a hierarchy of techniques (observations, simple statistical and complex numerical models), this study demonstrates that the MJO provides a source of predictability for the North Atlantic and particularly the adjacent shelf seas. This predictability originates from global-scale variations (e.g., MJO) and propagates to smaller scales through local processes (e.g., coastal upwelling). It has been shown that this cascade of scales can be utilized to downscale global S2S predictions of the ocean using regional high-resolution ocean models like GoMSS. In the future, this can be used as a tool to provide valuable, early information to decision makers.

## Figures and Tables

**Fig. 13:**
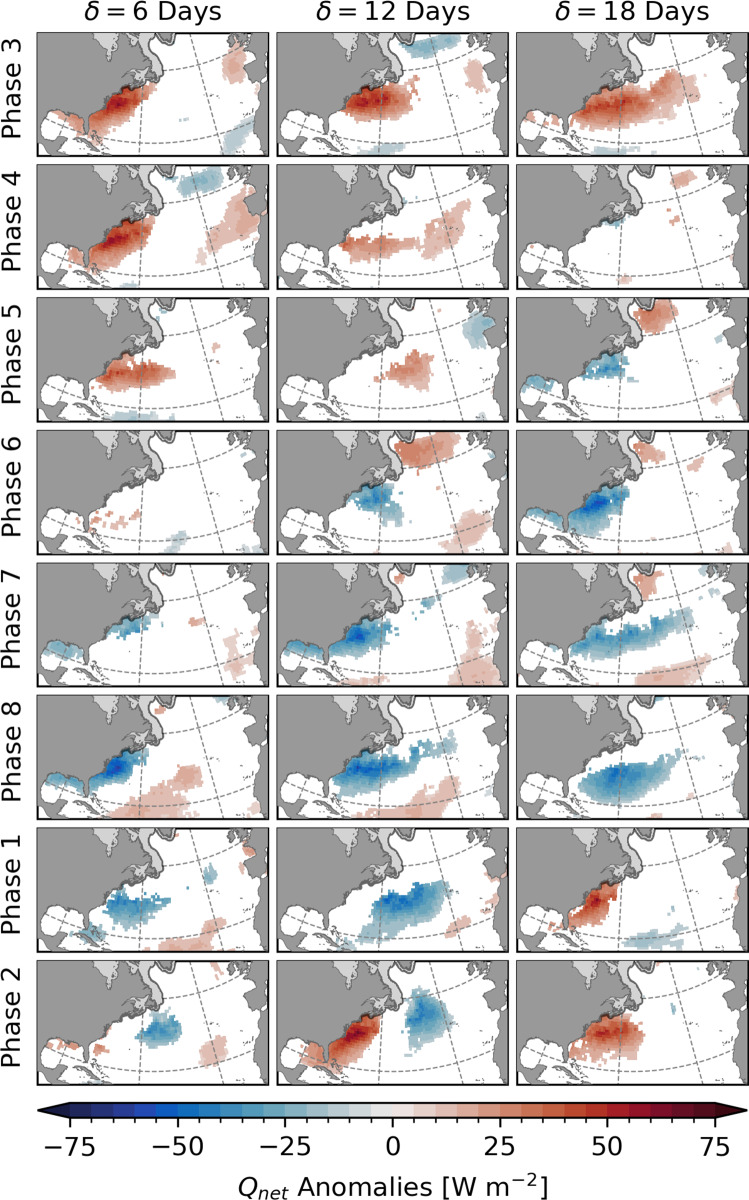
Composites of bandpass-filtered $Q_{\text {net}}$ anomalies for all MJO phases during winter when $A_\text {RMM}>$ 1. Each row represents a specific phase and the columns refer to lags $\delta =$ 6, 12, 18, and 24 days after that phase. All shown anomalies are statistically different from zero at the 10% significance level. Shaded areas near the coast show the climatological sea ice cover

## Data Availability

The RMM index is available at the Australian Bureau of Meteorology (http://www.bom.gov.au/climate/mjo/). The OISSTv2 dataset is available at the NOAA Physical Science Laboratory (https://psl.noaa.gov/data/gridded/data.noaa.oisst.v2.html). The OAFlux dataset is available at https://oaflux.whoi.edu/. The code and data required to configure and run the GoMSS model are publicly available: NEMO source code (https://www.nemo-ocean.eu/), NCEP CFSR data for surface boundary forcing (https://rda.ucar.edu/datasets/ds093.2/), GLORYS12v1 for open boundary conditions (https://data.marine.copernicus.eu/product/GLOBAL_MULTIYEAR_PHY_001_030), and FES2004 for tidal boundary forcing (https://www.aviso.altimetry.fr/en/data/products/auxiliary-products/global-tide-fes.html).

## Appendix: Net air–sea heat flux composites

Composites of net air–sea heat flux anomalies with respect to the MJO were computed using Eq. (7) by replacing the SST with bandpass-filtered $Q_{\text {net}}$ anomalies from the OAFlux dataset. The statistical significance of the heat flux composites was assessed using the moving-blocks bootstrapping method described in Sect. 3.

Figure 13 shows the spatial structure of $Q_{\text {net}}$ composites at lags $\delta =$ 6, 12, and 18 days (columns) for all MJO phases (rows). Only anomalies statistically different from zero at the 10% significance level are shown. Positive values refer to increased heat flux from the atmosphere into the surface ocean.

Focusing on the response to phase 3, it can be seen that a strong positive heat flux anomaly occurs along the eastern seaboard of North America and in the eastern Gulf of Mexico at lag $\delta$ = 6 days. This anomaly persists for several days, most notably north of the Gulf Stream region. At $\delta =$ 18 days after phase 3, the positive anomaly is slightly weaker, but more widespread, covering large parts of the western North Atlantic. Generally, the strongest anomalies can be observed in the Gulf Stream Extension Region.

The spatial pattern of net heat flux composites with respect to MJO phase 7 is, to first order, a mirror image of the anomalies for phase 3 at the same lags, but with reversed sign (Fig. 13). The centered anomaly correlation (ACC, e.g., Wilks 2011) between the composites for these two phases varies from $-0.56$ to $-0.85$ depending on the lag. The negative $Q_{\text {net}}$ anomaly in the northern Gulf of Mexico and along the North American east coast occurs at $\delta =$ 12 days after phase 7. This is expected since the convective dipole associated with the MJO during phase 7 is reversed compared to phase 3, therefore, leading to a similar response with opposite sign. However, the mirror imaging is not perfect. For example, the weak positive anomaly off the west coast of Africa “misses” its negative counterpart in the composite with respect to phase 3.

Overall, the composites show that there is a statistical relationship between the MJO and the net air–sea heat flux in the North Atlantic. The same composite analysis was conducted for the individual heat flux components in (6). The composites of incoming shortwave and outgoing longwave radiation are negligible and not statistically different from zero at the 10% significance level. This indicates that the $Q_{\text {net}}$ signal is primarily due to anomalies in sensible and latent heat flux (not shown).

Generally, the spatial structure of the heat flux and SST composites are similar. The difference in lags can be explained by the additional timescale $\delta _\text {O}$ for the SST to respond to the anomalous heating or cooling. This supports the hypothesis that the SST anomalies are driven by large-scale atmospheric perturbations in response to the MJO. However, the $Q_{\text {net}}$ anomalies are more spatially coherent than the SST composites suggesting that other processes, e.g., mesocale eddies or advection, and spatial variations in mixed layer depth, contribute to the spatial structure of the SST. Furthermore, the different spatial resolution of the datasets may also contribute to the discrepancy in the spatial scales of the composites.
